# A Genome-Wide Analysis of Promoter-Mediated Phenotypic Noise in *Escherichia coli*

**DOI:** 10.1371/journal.pgen.1002443

**Published:** 2012-01-19

**Authors:** Olin K. Silander, Nela Nikolic, Alon Zaslaver, Anat Bren, Ilya Kikoin, Uri Alon, Martin Ackermann

**Affiliations:** 1 Computational and Systems Biology, Biozentrum, University of Basel, Basel, Switzerland; 2 Department of Environmental Sciences, Eidgenössische Technische Hochschule (ETH) Zurich, Zurich, Switzerland; 3 Department of Environmental Microbiology, Eawag, Dubendorf, Switzerland; 4 Division of Biology, California Institute of Technology, Pasadena, California, United States of America; 5 Department of Molecular Cell Biology and Department of Physics of Complex Systems, Weizmann Institute of Science, Rehovot, Israel; Université Paris Descartes, INSERM U1001, France

## Abstract

Gene expression is subject to random perturbations that lead to fluctuations in the rate of protein production. As a consequence, for any given protein, genetically identical organisms living in a constant environment will contain different amounts of that particular protein, resulting in different phenotypes. This phenomenon is known as “phenotypic noise.” In bacterial systems, previous studies have shown that, for specific genes, both transcriptional and translational processes affect phenotypic noise. Here, we focus on how the promoter regions of genes affect noise and ask whether levels of promoter-mediated noise are correlated with genes' functional attributes, using data for over 60% of all promoters in *Escherichia coli*. We find that essential genes and genes with a high degree of evolutionary conservation have promoters that confer low levels of noise. We also find that the level of noise cannot be attributed to the evolutionary time that different genes have spent in the genome of *E. coli*. In contrast to previous results in eukaryotes, we find no association between promoter-mediated noise and gene expression plasticity. These results are consistent with the hypothesis that, in bacteria, natural selection can act to reduce gene expression noise and that some of this noise is controlled through the sequence of the promoter region alone.

## Author Summary

Many biological processes in a cell involve small numbers of molecules and therefore fluctuate over time. As a consequence, genetically identical cells that live in the same environment differ from each other in many phenotypic traits, including the expression level of different genes. Our aim was to identify types of genes with particularly low or high levels of variation (“noise”) and to understand molecular and evolutionary factors that determine noise level. Working with the bacterium *E. coli*, we analyzed the expression—at the single cell level—of more than 1,500 different genes. We found particularly low levels of noise in genes that *E. coli* needs to live and genes that this bacterium shares with many related taxa. This suggests that cellular functions that are particularly important for this organism evolved towards low levels of variation. In contrast to previous results with yeast, we find that genes that change their expression levels in response to environmental signals do not have high levels of noise. This suggests that there may be fundamental differences in how noise is controlled in bacteria and eukaryotes.

## Introduction

The phenotype of an individual is often considered to be a product of the individual's genotype and the environment in which it lives. However, significant phenotypic differences may exist between genetically identical individuals living in a homogeneous environment [1]–[7]. In the absence of genotypic differences or environmental cues, these differences often arise from random molecular processes during protein expression and development. In these cases, such variation is termed phenotypic noise. Although differences between individuals that are due to phenotypic noise are not encoded genetically, the level of phenotypic noise in a given gene may be subject to genetic control. One fundamental question is whether natural selection acts to control or promote phenotypic noise, and how organisms achieve this control.

It is well established that selection acts strongly on mean expression level [8]–[12]. Additionally, there is good evidence that selection can also act on the variation of gene expression, that is, on the level of phenotypic noise. Many studies with bacteria and other microorganisms have identified genes with exceptionally high levels of phenotypic noise, and several studies have provided possible adaptive explanations. Both theoretical [13]–[17] and empirical studies [18]–[21] have shown that increased noise and bistable gene expression can allow organisms to persist in fluctuating environments, and that selection may thus in some cases increase phenotypic noise. Other studies have shown that it can promote the formation of specialized subpopulations that engage in division of labor [5], [22].

However, there have been fewer studies on general patterns of gene expression noise, for example, across functional groups of genes. The best-established connection, and the only connection established for both eukaryotes and bacteria, is between mean expression level and variation in expression: strongly expressed genes have high levels of variation across cells [23], [24]. However, mean expression level does not fully determine variation: analyses in yeast have shown that when mean expression level is accounted for, gene expression noise exhibits certain strong patterns: for example, there is a positive association between gene expression noise and gene expression plasticity (i.e., variation in gene expression across environments) [24]; genes with TATA boxes exhibit high noise [24]; and those genes most critical for cell functioning exhibit lower levels of variation than other genes that are expressed at the same level [24]–[26]. This latter correlation is consistent with selection acting to decouple variation in expression from mean expression in order to decrease noise in important genes. However, this association is confounded by other correlations, such as the strong relationship between noise and expression plasticity.

There is no data addressing the question of whether functionally important genes exhibit lower levels of noise in bacteria: only one analysis of variation in gene expression has been performed in bacteria [23], which established that genes expressed at higher levels exhibit more extrinsic noise. This raises the question of whether these two properties can be decoupled, for example to lower noise in functionally important genes, even though these genes may be expressed at high levels.

Thus, although there is good evidence in yeast that genes important for cell functioning have lower levels of gene expression noise, the interpretation of this result as evidence of selection acting to decrease noise has been complicated by the association between expression plasticity and noise. Additionally, there have been no analyses of whether the decoupling of mean expression level and variation in expression exhibits such general patterns in bacteria. Here, we investigate this possibility, and whether such decoupling exhibits patterns on a general, genome-wide level.

In contrast to previous studies, which have examined protein expression noise, we carried out a comprehensive analysis of the noise conferred by the promoter regions alone in *E. coli*. Our goals were three-fold. First, we wanted to test whether the DNA sequence of the promoter region has a substantial and consistent effect on noise. Second, we asked whether differences in noise exhibit discernible patterns, for example across functional categories of genes. Finally, we assessed whether these patterns are consistent with selection acting to preventing or promoting phenotypic noise, or whether other explanations account equally well for the patterns we observe.

## Results

### Experimental system

We used an *E. coli* promoter library [27] consisting of 1832 strains, in which each strain carries a low-copy number plasmid (3–5 copies per cell [28], [29]) with an *E. coli* promoter region inserted upstream of a gene for a fast-folding green fluorescent protein (*gfp*). This library comprises about 75% of all *E. coli* promoters. The term ‘promoter region’ refers to the intergenic region between two open reading frames, together with 50–150 nucleotides of both the upstream and downstream open reading frame [27]. The mRNA that is produced consists of a transcriptional fusion between a short region of the 5′ end of the native mRNA, 31 bp that are identical for all promoters, and the open reading frame for GFP. A strong ribosome binding site (RBS) is located immediately upstream of *gfp*. As the 31 bp preceding the gfp start codon are identical for all constructs, effects from differences in the translation initiation rate should be minimal [30], [31]. Additionally, as approximately 90% or more of the mRNA sequence is identical for each construct, in most cases, differences in mRNA half-lives between constructs are likely to be small. The GFP variant is quite stable, so decreases in protein concentration occur primarily through cell growth and division. For the above reasons, differences in the mean concentration of cellular GFP for different promoters are most likely due to differences in transcription (see Text S1). However, in many instances the promoter region may affect mRNA half-life or translation dynamics, since it contains a fraction of the native open reading frame.

This experimental system removes several mechanisms that are likely to affect protein expression noise in the native context. Among these is the chromosomal context of the gene; the mRNA sequence content, affecting both mRNA half-life and translation; and the amino acid sequence, affecting protein degradation. In fact, the only variable among the constructs is the sequence of the promoter region. By definition, then, the effects on noise that we measure here are due to the promoter sequence alone. This experimental approach thus allows us to investigate whether and how the promoter sequence alone affects noise. Although this promoter-mediated noise contributes only partially to the total noise exhibited by a protein, it may play an important role, which we investigate here; later we use data on protein noise to explore other factors that contribute to affecting protein expression noise.

### The nucleotide sequence of the promoter region is a consistent determinant of phenotypic variation

To quantitatively measure variation in gene expression from each promoter, we grew a clonal population of each strain, and used flow cytometry to measure the GFP concentration in approximately 100'000 individual cells from each population. For each strain, we extracted a small gated subset of cells (Figure S1; see Methods). This gating has the effect of minimizing extrinsic variation due to physiological differences among cells, such as cell cycle timing, slow growth, or other physiological stresses (see Text S1). For each of 1832 strains containing a promoter region from *E. coli*, we measured the mean and variance in fluorescence. 1522 of these yielded measurements significantly above background (GFP vector lacking a promoter; see Methods). We use the data from these 1522 promoters for the remainder of our analyses.

The mean and variance of fluorescence are highly repeatable measurements; when they were assessed for independent cultures, repeated measurements were extremely accurate (r^2^ = 0.998 and 0.91, for mean and standard deviation, respectively). This repeatability existed even when the cultures were grown in different laboratories, measured on different flow cytometry machines, and when different methods were used to filter events (r^2^ = 0.92 and 0.51 for mean and standard deviation, respectively; see Methods and Figure S2). Mean fluorescence levels varied over almost 3 orders of magnitude, qualitatively similar to the variation in mRNA levels observed in other studies [23]. Comparing our data on mean fluorescence level with published quantitative data, we also find that our data set correlates well with measured transcript levels, and is thus likely to capture an important aspect of mRNA transcription (see Text S1).

**Figure 1:**
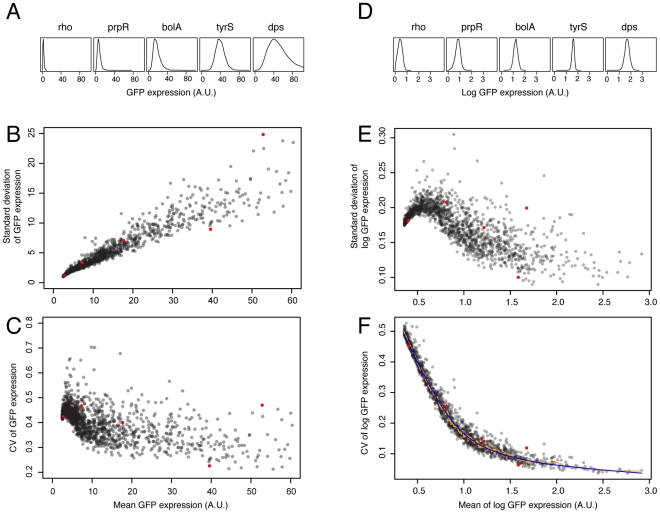
Dependence of variation in mRNA expression on mean mRNA expression level and derivation of a noise metric. A. The observed variance in mRNA expression increases with increasing mean expression level. Shown are five promoters with various levels of mean and variance in expression (from left to right: rho transcription termination factor; *prpR* transcriptional dual regulator, *bolA* transcriptional dual regulator; tyrosyl-tRNA synthetase; and *dps*, an iron sequestration and DNA damage protein). B. The expression level and observed standard deviation for all 1522 promoters used in the analysis. The genes shown in panel A are highlighted in red (the left-most red dot is *rho*, the right-most dot is *dps*). C. The coefficient of variation decreases initially with increasing expression, but plateaus at higher expression levels. D–F. Analogous histograms and graphs to those shown in panels A–C), but calculated from log-transformed data. As discussed in the text, our focus is on variation in expression; we thus derived a measure of variation in mRNA expression that is independent of the mean level of mRNA expression, and any measurement artifacts associated with changes in the mean. This allows us to test whether mean and variation in expression can be decoupled due to selection or changes in the promoter sequence. The noise metric is the vertical deviation from a smooth spline (blue) calculated from the running median (orange) of mean log expression level versus the CV of log expression. The slight decrease in CV at low expression levels (panels C and E) is because fluorescence values lower than one cannot occur. Thus, for weakly expressed genes, the distribution specifying the variation in expression levels is truncated at one, decreasing the CV.

We find a strong dependence of variation in expression on mean expression level for any particular promoter (Figure 1), as has been observed previously [23], [24], [32]. Because the primary effect of selection on gene expression occurs as stabilizing selection on mean expression level [8]–[11], and mean and variation are closely coupled, we use a metric that decouples variation in expression from mean expression level. Modifying the method outlined by Newman et al. [24] we measured noise as the vertical deviation from a smoothed spline of mean log expression versus the coefficient of variation in log expression for all promoters in the library (see Methods; Figure 1F; Text S1; Dataset S1). When describing our findings, the term ‘phenotypic noise’ or ‘noise’ always refers to this metric in which variation is corrected for mean expression; such a measure allows us to assess whether variation in gene expression is controlled independently of the mean.

We emphasize that we use the term ‘noise’ to refer to relative differences in variation when mean expression level is controlled for. Thus, it is a qualitative measure, and for this reason we emphasize comparative results of relative differences in promoter-mediated variation; also for this reason, we restrict our statistical analyses to non-parametric tests. We refer to this measure as ‘noise’ because it is a reflection of differences between cells that are likely to arise from stochastic events, but it is not a quantitative measure of the frequency or effect of those events. In addition, because we have functional data for genes only, and not promoters, when we refer to the noise of a ‘gene’ or the functional category of a ‘promoter,’ we are referring only to the gene that lies directly downstream of the promoter, unless otherwise specified.

When we calculate this noise metric for the entire library of promoters, we find excellent repeatability, even in different culture conditions. The correlations range from 0.50 (Spearman's rho) when using data from different labs, to 0.58 when using data collected in independent experiments in the same lab (Figure S3). These are lower limit estimates of repeatability, as in each of these comparisons different culture conditions were used (see Methods). The repeatability of the noise metric implies that each promoter sequence has a consistent effect on variation in expression: thus, as suggested above, there are characteristics inherent to each promoter that result in different levels of noise.

Noise in gene expression consists of different components [33], [34], and our experimental system mostly reports one of them, promoter-specific extrinsic noise. Since the promoter-gfp construct resides on a plasmid with several copies, the cellular GFP concentration is the sum of the contributions from individual promoters. Intrinsic noise – variation generated at the level of one single promoter – is therefore decreased. In addition, because the GFP protein has a longer half-life than mRNA, the sensitivity of these noise measurements to intrinsic noise events in transcription is decreased [35]. Finally, fluctuations in plasmid number, which are expected to increase noise in all strains equally, may decrease the sensitivity of this system.

The noise that we measure is thus a qualitative and relative indication of the amount of promoter-specific extrinsic transcriptional noise [33], [34]. If we measure high levels of noise in a protein controlled by a particular promoter, most likely this occurs because transcription from this promoter is controlled by factors (or regulatory networks) having higher noise, or that this promoter is more sensitive to global extrinsic noise factors (e.g. variations in polymerase numbers) than other promoters. This experimental system is thus useful to examine extrinsic promoter-mediated noise on a genome-wide scale, and to ask how the level of extrinsic noise differs among promoters.

Even though, as discussed above, our plasmid-based system only captures some aspects of gene expression, we find that it gives similar results to chromosomally integrated systems in both mean and variation of expression. We measured the mean and variation in expression for nine chromosomally integrated promoter-*gfp* fusion constructs [36], and found that both the mean and CV correlate well with the values that we find for the plasmid-based system (rho = 0.85, p = 0.006; rho = 0.77, p = 0.016 for mean and CV, respectively; see Text S1 and Figure S4).

### Promoters of essential and conserved genes have lower levels of noise

Given that the promoter sequence alone has a consistent influence on mRNA expression and noise levels (above; Figure S3), this raises the question of whether these levels of noise systematically differ for different classes, or types, of promoters. One broad division that can be made is between promoters that drive the expression of essential genes and those that drive the expression of non-essential genes (we define a gene as essential if its deletion yields an inviable genotype in rich media [37]). We used data for 118 promoters that lie directly upstream of essential genes or operons [38] that contain at least one essential gene, out of 1456 promoters for whose downstream genes we have information about essentiality. We find that promoters of essential genes exhibit significantly lower levels of noise than other promoters: of the genes with the lowest level of noise (first quartile), 13.1% are essential; of the genes with the highest level of noise (fourth quartile), only 2.9% are essential (p = 1.0e-6, Wilcox rank sum test). This difference is not driven by any mechanisms relating to mean expression levels, since our measure of noise corrects for this. Thus, the promoter regions of genes that are essential in the laboratory environment have evolved such that essential genes have lower noise levels.

**Figure 2:**
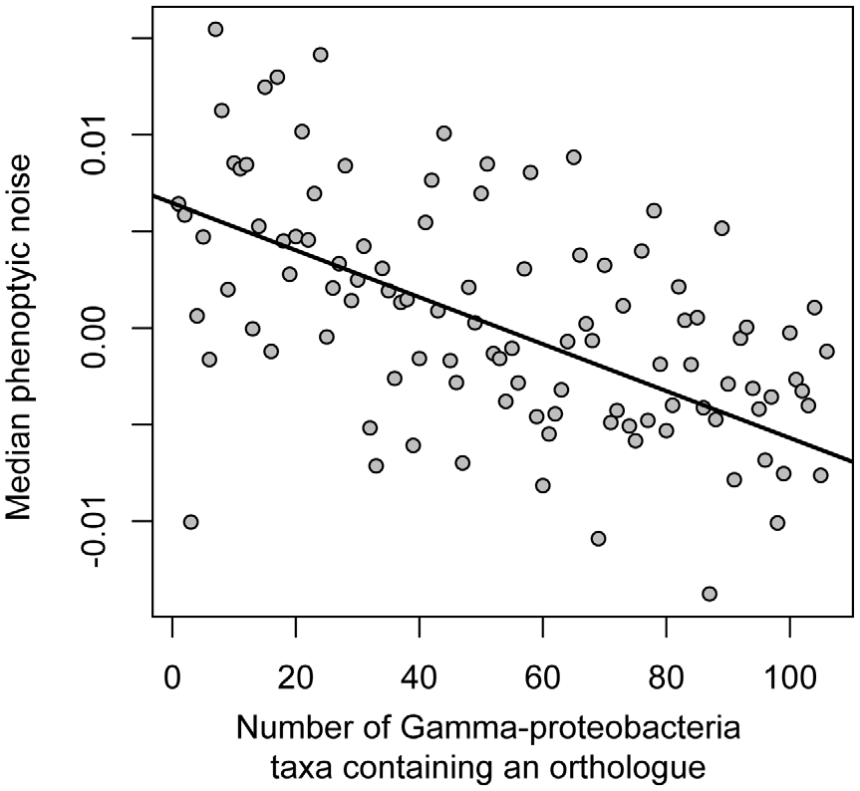
Noise in gene expression is dependent on the functional importance of the downstream gene. Conserved non-essential genes exhibit less noise. Conservation is calculated as the number of gamma-proteobacterial taxa in which an orthologous gene copy is present. Promoters were binned according to the number of taxa in which an orthologue was found; the relationship is highly significant (for an unbinned analysis, Spearman's rho = −0.19, p = 7.2e-12, n = 1350 (Figure S5)). A nonparametric linear fit using Thiel's method [71] is shown in black.

Essentiality in the laboratory is an incomplete and potentially biased measure of a gene's importance in the natural environment. We thus also looked at gene conservation, which may capture additional aspects of functional importance [39], [40]. Considering non-essential genes only, we found a negative relationship between noise and functional importance: non-essential genes that have high levels of conservation in the gamma-proteobacteria clade (of which *E. coli* is a member) have promoters conferring low levels of noise (Spearman's rho = −0.19, p = 7.2e-12, n = 1350; Figure 2 and Figures S5 and S6). Furthermore, this relationship between conservation and expression noise exists within functional categories: it does not depend on broad differences in conservation between genes of different function, for example between genes involved in RNA production (expected to be more conserved and less noisy) versus those involved in carbon metabolism (expected to be less conserved and more noisy; Figure S7).

Together with the above data on essential genes, this suggests that the promoter regions of functionally important genes confer low levels of noise; given that the major effect of promoter sequence on protein level occurs through mediating transcription, this decrease in noise likely occurs through the control of transcriptional processes. The transcriptional regulation of some bacterial genes has been shown to be constructed such that increased noise is a result [41]; the data here suggest that on a genome-wide basis there is a tendency for functionally important genes to be controlled by less noisy transcriptional processes, that this trend extends beyond essential genes to conserved, non-essential genes, and that this trend persists within functional categories of genes.

### Evolutionary history is not a primary driver of the decreased noise in promoters of essential and conserved genes

There are several possible explanations for the low levels of noise observed in essential and highly conserved non-essential genes, two of which we discuss here (we explore a third explanation in the following section; however, this list is not exhaustive). First, it is possible that essentiality and gene conservation are good descriptors of the functional importance of a gene, and that selection has acted to decrease noise in such genes. This has been the explanation put forth in previous analyses. A second possible explanation is that low noise levels are difficult to evolve, and as conserved and essential genes have also spent more evolutionary time in a particular genome than non-conserved genes, selection has had more time to minimize noise in these genes. Either of these explanations could result in conserved and essential genes having lower noise. However, the results of our analysis suggest that the second explanation is less likely, for the following reasons.

First, the correlation between gene conservation and noise exists even for genes that have been acquired very distantly in the past. We looked for an association between functional importance and noise considering only genes acquired before the divergence of the *E. coli* lineage from alpha-proteobacteria (approximately 2.5 billion years ago [42]). These genes have had ample time for noise minimization. Thus, if the time a gene spends in a particular genome is a strong determinant of noise, there should be no relation between conservation and noise in this set of genes, as all have spent at least 2.5 billion years in the *E. coli* lineage. However, the correlation between conservation and noise within these anciently acquired genes remains strong (Spearman's rho = −0.23, p = 2.8e-4, n = 249). That the amount of noise minimization is related to the level of evolutionary conservation (functional importance) even in anciently acquired genes strongly suggests that the time that a gene spends in an organism has little to do with the level of noise it exhibits.

Second, although horizontally transferred genes are generally enriched for genes of lesser functional importance, many genes important for cell functioning have been horizontally transferred (e.g. some ribosomal genes). Among genes that have been recently horizontally transferred into *E. coli* [43], strongly conserved genes have lower levels of noise (correlation between noise and conservation: Spearman's rho = −0.22, p = 6.9e-3, n = 221 for genes transferred after the split of *E. coli* from *Haemophilus*; Spearman's rho = −0.25 p = 4.8e-4, n = 171, for genes transferred after the split of *E. coli* from *Buchnera*). When we consider very recent horizontal gene transfers the negative correlation remains (Spearman's rho = −0.16, p = 0.23, n = 65 for genes transferred after the split of *E. coli* MG1655 from *E. coli* CFT073). Although this correlation is not significant, there are only a small number of recently transferred genes, and these vary little in their levels of evolutionary conservation, decreasing the explanatory power of this variable. Given that the nucleotide divergence between MG1655 and CFT073 strains is approximately 2% [44], finding a negative correlation of similar strength (−0.16 vs. −0.19 for the entire data set) is notable.

Thus, the relationship between functional importance and noise does not appear to be related to the time that a gene has spent in a genome. The latter result also implies that the decreased noise observed in functionally important genes, if due to selection, can occur via a small number of mutations. Alternatively, it is possible that features of the promoter that influence noise act independently of the genetic background, so that genes retain characteristic levels of phenotypic noise even when horizontally transferred. We do find some support for this latter hypothesis: promoters of very recently horizontally transferred genes (ORFan genes; e.g. [45]) do not exhibit higher levels of noise than other promoters (Wilcox rank sum, p = 0.69, n = 37).

### There is no evidence that noise is an unavoidable consequence of selection for expression plasticity

Our results, showing that functionally important genes exhibit lower gene expression noise, is consistent with the hypothesis that selection has acted to decrease noise in genes important for cell function. However, many other factors may potentially play a role in determining noise. A crucial determinant of noise in gene expression may be in how the gene is regulated: genes that exhibit large expression plasticity, meaning that they can undergo strong repression or activation across different environmental conditions, might be controlled in ways that makes them intrinsically more noisy. A very strong association between expression plasticity and noise has been found previously in yeast [24]–[26].

To investigate whether there is a similar association between noise and expression plasticity in *E. coli*, we gathered data on changes in gene expression across 240 pairs of environmental conditions [46]. For each pair of conditions, gene expression changes are expressed as the log ratio of expression in one condition relative to a reference condition; the value is positive for genes that increase their expression, and negative for genes that decrease their expression under the respective environmental condition. For each gene, we calculated the median of the absolute values of the expression changes. This value, which we term the expression plasticity, is high for genes whose expression frequently varies between two conditions, and low for genes whose expression is usually constant between two conditions, regardless of whether this occurs through repression or activation, or the nature of the reference condition.

Surprisingly, we found no significant association between noise and expression plasticity in *E. coli* (Spearman's rho = 0.030, p = 0.27, n = 1354). It is possible that this correlation exists only in some growth conditions, and that these types of conditions are under-represented in the dataset. To account for this possibility, we grouped the condition pairs by their similarity in expression changes into 18 clusters, calculated the median of the absolute values of the expression changes, and again found no significant correlation (Spearman's rho = −0.002, p = 0.94, n = 1354). Performing a similar analysis for yeast yields a significant positive relationship between expression plasticity and noise (data from [47]; unclustered analysis: Spearman's rho = 0.22, p = 7e-26, n = 2479). Although the lack of a correlation in *E. coli* could be driven by differences in data quality, this is not a likely explanation (see Text S1 and Figure S8).

Together, these data suggest that in yeast, a substantial fraction of gene expression noise might be a consequence of requiring dynamic control of gene expression [26]. However, in *E. coli*, high gene expression noise is not an unavoidable consequence of genes having high expression plasticity. Further supporting this conclusion is the association between functional importance and expression plasticity in *E. coli*: essential and conserved genes are the most dynamically regulated: 42% of essential genes are among the most dynamically regulated genes (within the top quartile), while only 13% are among the least dynamically regulated (bottom quartile) (p = 5e-6, Wilcox rank sum for essential versus non-essential genes; Spearman's rho = 0.19, p = 1.1e-11, n = 1209 for the correlation between expression plasticity and conservation). Despite this, promoters of essential and conserved genes exhibit the lowest level of noise. Thus, in *E. coli*, there does not appear to be a constraint preventing promoters with high expression plasticity from having low noise. In contrast, there is a strong positive correlation between expression plasticity and noise in yeast, suggesting that for many genes, such a constraint may exist. Because essential genes in yeast have low expression plasticity (see Text S1), the previous finding that essential yeast genes exhibit low levels of noise might be a consequence of this association between expression plasticity and noise.

### Functional classes differ in their levels of noise

**Figure 3:**
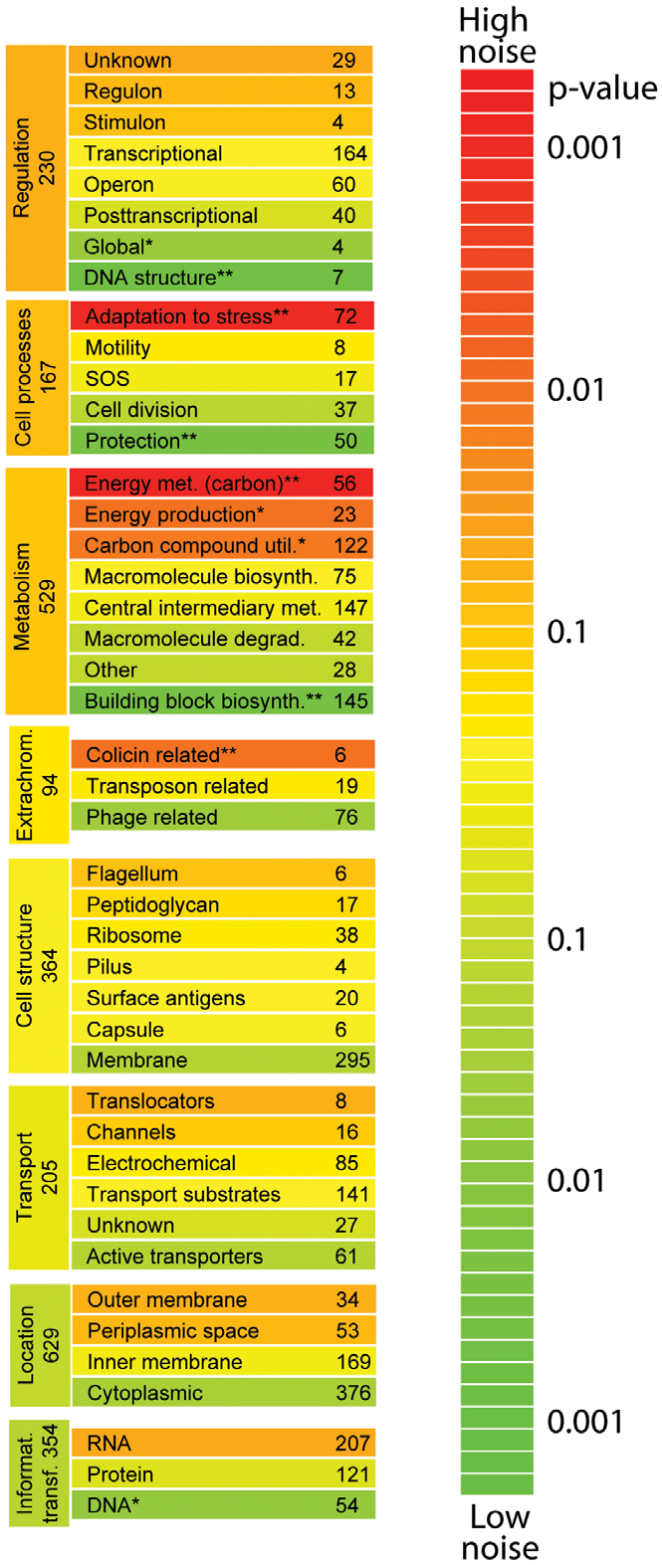
Noise in gene expression is related to the specific functional role. Genes in different functional categories exhibit high or low levels of noise. We considered eight of the major categories delineated by MultiFun (metabolism, information transfer, regulation, transport, cell processes, cell structure, location, and extra-chromosomal origin) [48]. Within each of these categories, we asked whether there were consistent differences in the amount of noise exhibited by genes of different function. Major categories and subcategories are ranked by the amount of noise exhibited by genes in that category; within each major category, subcategories are colored relative to the average amount of noise exhibited by all genes in the major category. The color indicates the probability of the null hypothesis (that genes in a given subcategory have the same level of noise as genes in other subcategories; two-sided Wilcox rank sum test). Two stars indicates that the subcategory exhibits a significantly higher or lower level of noise than other subcategories after correcting for multiple comparisons; one star indicates that the subcategory exhibits a higher or lower level of noise with p<0.05. Regulation is the only major functional category that exhibits higher noise, although this result is of only marginal significance.

We looked in more detail at how specific functional aspects relate to gene expression noise. We grouped genes according to the categories outlined by MultiFun [48], and found substantial differences between genes having different functional roles (Figure 3). Relatively low levels of noise were exhibited in genes involved in DNA structure (i.e. methylation, bending, and super-coiling) and building block synthesis (biosynthesis of amino acids, nucleotides, cofactors, and fatty acids). Low levels of noise in such housekeeping genes might be expected, given that normal cellular activities are probably compromised if these proteins are too abundant or not abundant enough, as has been suggested previously [49], [50]. We also observed particularly low levels of noise in genes involved in protection (from radiation, cell killing, drug resistance, or for detoxification). Finally, promoters annotated as having binding sites for σ32 (control of heat shock genes) have significantly lower levels of noise; several transcription factors are also associated with low noise (Table 1).

**Table 1:** Sigma factors and transcription factors associated with genes exhibiting low or high levels of expression noise.

Transcription factor	Number of target genes	Noise level	p-value (two-sided Wilcox rank test)
MetJ	10	low	0.0016
σ32	64	low	0.0052
CpxR	18	low	0.011
ArgR	16	low	0.017
NarP	6	high	0.039
TrpR	6	high	0.029
GadX	10	high	0.027
Fnr	71	high	0.015
Hns	43	high	0.0099
GadW	5	high	0.0089
NarL	19	high	0.0086
IhfA/B	48	high	0.007
σ38	85	high	6.7e-7

We analyzed all factors listed in RegulonDB that regulate five or more targets (6 sigma factors and 43 transcription factors in total). All factors with p-values less than 0.05 (uncorrected for multiple tests) are shown.

Particularly high levels of noise are primarily found in genes involved in two functional groups: energy metabolism of carbon sources (e.g. glycolysis, the pentose phosphate shunt, fermentation, aerobic respiration), and in adaptation to stress (osmotic pressure, temperature extremes, starvation response, pH response, desiccation, and mechanical, nutritional, or oxidative stress). Finally, promoters with binding sites for σ38 (control of starvation and stationary phase genes) exhibit higher levels of noise than promoters containing binding sites for other sigma factors; several transcription factors were also associated with higher noise levels (Table 1).

As the above analysis implied that high levels of noise are not simply a consequence of having high expression plasticity, the differences in noise between categories is consistent with differential selection (although other factors may also be responsible). For example, one possibility is that some genes exhibit high levels of noise due to an absence of selection (such that drift dominates the evolutionary process), in contrast to the majority of genes in the genome. A second possibility is these genes have experienced selection for high levels of noise. Variation in resource utilization between cells can sometimes increase the growth rate of clonal populations [19], [51] by promoting the utilization of carbon sources that become newly available. Similarly, noise in genes involved in adaptation to stress could allow genotypes to persist under conditions where stressors appear quickly [13], [52], [53]. Alternatively, genes with high noise may also be constrained in their ability to evolve lower noise due to trade-offs with other functions that we have not measured. These results thus generate explicit and testable hypotheses about the possible adaptive functions of increased noise in gene expression.

### Protein expression noise is controlled through both transcriptional and post-transcriptional mechanisms

Our focus until now has been on how the nucleotide sequence of a promoter alone controls phenotypic noise in a plasmid-based context. Noise at the level of protein is possibly controlled through additional mechanisms acting at the post-transcriptional level. To include these mechanisms into our analysis, we used data from a recent study that measured variation in protein numbers between cells for a large number of the protein coding genes in *E. coli* [23]. This study was based on translational fusions of protein coding genes with YFP in the native chromosomal context. Using approximately 1'000 of these constructs, the authors used microscopy to measure the mean and variation in protein number per cell. This study thus provides us with information on the sum of intrinsic and extrinsic noise that occurs through both transcriptional and post-transcriptional processes.

Using this dataset, we quantified protein expression noise in an analogous manner as for our data, removing genes with very low expression levels and correcting for mean protein expression level. Again, this decouples mean protein expression level from variation in protein expression. We find a significant but weak correlation between protein noise in this dataset and gene expression noise in our own (Spearman's rho = 0.12, p = 0.02, n = 334). A primary reason for this low correlation may be that the noise in protein expression was measured during late exponential phase, while we measured during early exponential phase growth (see Text S1). We find that, similar to the pattern observed for promoter-mediated noise, essential and conserved genes have low protein expression noise (Wilcox rank sum, p = 3e-4, n = 116 essential genes; Spearman's rho = −0.21, p = 7.0e-9, n = 645 non-essential genes). Using variation alone as a metric of noise, without the correction for mean expression level, gives the opposite result: essential genes have significantly higher levels of variation [23], as they are expressed at higher levels, and variation is strongly positively correlated with mean expression. Finally, corroborating the lack of correlation between promoter-mediated noise and expression plasticity, protein expression noise and plasticity exhibit no significant correlation (rho = 0.052, p = 0.16, n = 724).

We find that post-transcriptional processes play a role in controlling protein expression noise: genes with high protein expression noise have slightly higher rates of translation initiation (Spearman's rho = 0.17, p = 3.3e-6, n = 730; computational predictions of ribosomal initiation rates from [30], [54], and slightly longer mRNA half-lives [55] (Spearman's rho = 0.15, p = 4.4e-5, n = 689). This is consistent with the idea that intrinsic noise in post-transcriptional mechanisms has a significant effect on total noise, as theoretical models have suggested [34], [56]–[58]. However, the extent to which the cell actually employs these mechanisms has remained unknown. The data here suggest that these mechanisms affect the noise levels of many genes in *E. coli*. If this association has occurred through selection, this implies that although these mechanisms are quite costly for the cell [59], the advantage of controlling intrinsic noise outweighs the energetic costs that it imposes.

## Discussion

We have shown here that by using a simple plasmid based system that different promoters consistently confer different levels of phenotypic noise. In particular, we find that functionally important genes have promoters that confer lower levels of gene expression noise, and certain functional categories are enriched or depleted for promoters that confer high noise. The noise metric we use accounts for mean expression level, so these patterns are not due to differences in expression levels between essential and non-essential genes, or to characteristics related indirectly to expression level (for example, systematic differences in cellular stress levels due to GFP). Furthermore, these noise characteristics appear to extend across different growth conditions, as promoter-mediated noise is similar during growth in non-stressful (arabinose and glucose) and stressful (low-levels of antibiotic) conditions (see Figure S3).

We have excluded several confounding factors from the association between noise and functional importance, including the age of the gene and the association with expression plasticity. The lack of association between promoter sequence and expression plasticity is surprising, given the strong relationship that has been observed in yeast [24], and that promoter sequence is a strong determinant of transcript level (see Text S1). The low noise of promoters of functionally important genes is consistent with the hypothesis that natural selection acts to control against variation in proteins that are important for cellular functioning [60]. However, it is important to emphasize that we cannot exclude other factors being responsible for this pattern.

We cannot yet determine the level at which the effects of promoter-mediated noise control extend to the protein level. Processes downstream from transcription may have significant effects on noise, and might sometimes overwhelm the effects arising on the transcriptional level. The association that we find between promoter-mediated noise and protein noise suggests that in many cases, transcriptional noise does correspond with the noise observed further downstream. However, we cannot say how strong this association is for all genes.

As our noise metric largely excludes both intrinsic noise and global extrinsic noise, these results suggest that promoter-mediated noise is systematically reduced in functionally important genes through gene-specific mechanisms. Thus, it seems that the regulatory inputs for these promoters have evolved to minimize noise. This has been shown previously for single regulatory networks [61]; here we show that it also appears to occur for many different genes. In addition to promoter-mediated control of noise, we find that proteins that exhibit low levels of noise have short mRNA half-lives and low rates of translation initiation. Although previous work has shown that variation in expression is strongly positively associated with mean expression level [23], the data here show that these two characters can be uncoupled, so that transcriptional noise can be controlled independently of the mean, and that this uncoupling is stronger for some types of genes (those that are functionally important) than others.

Although it has been hypothesized previously that functionally important genes have been selected to exhibit low levels of noise [62], it has been difficult to unambiguously show this. In particular, it has been difficult to separate the effects of expression plasticity and low noise, as all previous studies connecting noise and functional importance have been in yeast, where this association is quite strong [24]–[26] (see Text S1). The data shown here provide evidence that in *E. coli*, these two characteristics are unconnected.

**Figure 4:**
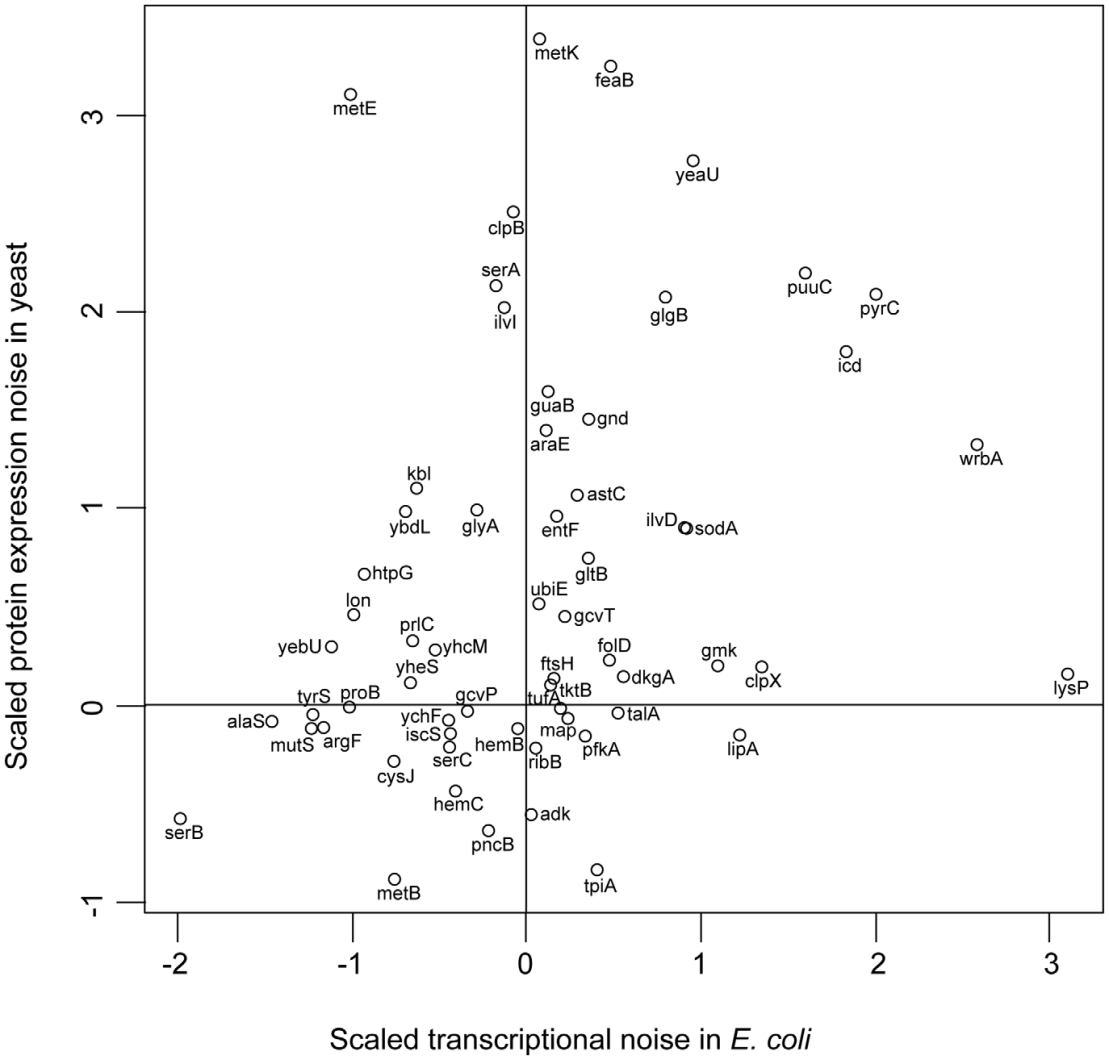
*E. coli* and yeast orthologues have similar noise levels. Orthologues in *S. cerevisiae* were determined through a reciprocal best-hit analysis for all the genes in *E. coli*. The number of reciprocal best-hit orthologues depended on the e-value cut-off that was set; this plot shows all orthologues with an e-value lower than e-35 (rho = 0.31, p = 0.015, n = 60). The data on yeast noise are from [24]; noise values for both *S. cerevisiae* and *E. coli* have been scaled such that the mean is zero and the standard deviation in noise is one.

In eukaryotes, one of the dominant regulatory mechanisms associated with transcriptionally noisy genes is chromatin structure (noisy genes tend to contain TATA boxes and are frequently regulated by SAGA [21], [24], [63]). A corollary of this is that in yeast there is a strong association between noise and expression plasticity, as dynamically regulated genes are often associated with chromatin remodeling factors. Much of this noise is thought to arise because of the two step process inherent in eukaryotic transcription, in which initial access to the DNA occurs through relaxation of histone binding, followed by transcription factor and polymerase binding [64]. Homologous mechanisms do not exist in bacterial systems; this may fundamentally affect the correlation between noise and expression plasticity. Despite these mechanistic differences, we do find a significant positive correlation between the promoter-mediated noise in *E. coli* genes and protein expression noise in their *S. cerevisiae* orthologues (rho = 0.31, p = 0.015, n = 60; Figure 4). Thus, although these organisms might differ in the mechanisms affecting gene expression noise, genes of similar function do exhibit similar levels of noise. However, protein expression noise, as calculated from [23] exhibits no correlation with gene expression noise in *S. cerevisiae*.

The data presented here show that: (1) For many genes, the promoter region of a gene controls noise in a consistent manner; (2) Functionally important genes are controlled such that noise is decreased; (3) The lower noise observed in functionally important genes does not appear to result from these genes having been present in the genome for a longer period of time; (4) There is no correlation between the noise conferred by a promoter and the expression plasticity of mRNA expression that is controlled through that promoter. In particular, this latter observation implies that there may be fundamental differences between the mechanisms giving rise to phenotypic noise in bacterial versus eukaryotic systems.

## Methods

### Strains

All strains have been described previously [27]. Briefly, each strain in the library contains a plasmid with a ‘promoter region’ cloned upstream of a fast-folding GFP. These promoter regions consist of an intergenic region, together with 50–150 bp of the upstream and downstream genes. The inclusion of part of the upstream and downstream open reading frames ensures that the majority of transcriptional control elements are contained in the construct. The library contains all K12 intergenic regions longer than 40 bp. We note that although the system is plasmid based, copy-number variation is relatively low. The plasmid contains an SC101 replication origin, for which segregation is tightly controlled [29]. For this reason variation in plasmid number per cell is expected to be less than under a binomial distribution, although variation in plasmid numbers will contribute additional extrinsic noise.

The strains with chromosomal integrations of the promoter-GFP fusions have been described previously [36]. Briefly, the promoter-GFP fusions were cloned and inserted into the attTn7 locus using a delivery plasmid containing a multiple cloning site surrounded by the terminal repeats of Tn7 [65].

### Growth conditions, sample preparation, and flow cytometry

All strains were grown in minimal media (M9) supplemented with 0.2% arabinose. Overnight cultures grown in same media were diluted 1∶500 and allowed to grow to mid-exponential phase at 37°C, shaken at 200 rpm. The cells were incubated with Syto red 62 (Molecular Probes) to stain the chromosome. The filters used for cytometry were 488/530+/−15 for GFP and 633/660+/−10 for the nucleic acid staining. In calculating the repeatability of the noise metric (Figure S3), two additional growth conditions were used: M9 supplemented with 0.2% glucose, and M9 supplemented with 0.2% glucose and 2.5 ng/ml ciprofloxacin.

### Filtering of data

The data were collected from a culture containing cells in different physiological states and quality. To minimize heterogeneity driven by these processes, we selected a small subset of cells with minimal CV. For the majority of promoters, the CV of the population was minimized between 5,000 and 10,000 cells, although gating had only a minimal effect on CV, decreasing it by 10–20% at most. Larger values than this generally contained cells of differing size and complexity, affecting the variance in fluorescence; smaller values contained too few cells to be a reliable indicator of the population. Thus, for all promoters, fluorescence data for 100,000 cells was collected and this data was subsequently filtered so that the fluorescence data from only 10,000 cells were analyzed further. These data were exported into text files and analyzed using the R statistical framework [66] (the raw data is available at http://mara.unibas.ch/silander.html).

The filtering process occurred in one of two ways. For the majority of the analysis, it occurred as follows: (1) the first 1000 acquisition events were excluded to minimize inaccuracies in fluorescence measurements resulting from sample crossover and initial inaccuracies in measurements that we observed; (2) extreme outliers (all cells with red fluorescence values below ten and GFP values of one or less) were removed; (3) to enrich for cells in similar physiological states and stages of the cell cycle, for each promoter, a kernel density was fitted to the log red fluorescence data (indicative of the amount of nucleic acid in the cell), with Gaussian smoothing in which the density was estimated at 512 points using the method of Silverman for bandwidth selection [67]. The maximum value of this kernel density was determined, and 10,000 cells were selected from a symmetrical interval around this peak (see Dataset S2 for simplified code). This number of cells minimized the variation in GFP signal due to external influences (Figure S2), while still allowing us to sample a large number of cells. The mean, median, and standard deviation for this population of cells were then calculated.

For secondary confirmation of previous measurements, events were filtered on the basis of FSC and SSC alone: (1) again, the first 1000 acquisition events were excluded; (2) extreme outliers (all cells with SSC, FSC or GFP values of one or less) were removed; (3) a bivariate normal was fit to the log FSC and log SSC values, and values outside of two standard deviations were removed (cellular debris); (4) to enrich for cells in similar physiological states and stages of the cell cycle, a 2 d kernel density was fitted to the FSC and SSC data. The maximum value of this kernel density was determined, and 10,000 cells were selected from an elliptical gate around this point, oriented by the covariance between FSC and SSC (Figure S1). This gating was performed using the flowCore package [68]. Again, the mean, median, and standard deviation for this population of cells were calculated.

Several promoters gave rise to distributions that appeared to be either bimodal or have extremely high variances. The promoters having the highest CV (>0.6), and all promoters exhibiting a bimodal expression pattern were reanalyzed by restreaking for single colonies and measuring fluorescence a second time. We found that for all promoters exhibiting bimodal patterns, the bimodality disappeared upon restreaking to obtain a single clone; a previous analysis of protein levels in *E. coli* cells confirms the rarity of bimodal distributions [23]. We thus concluded that the bimodal distributions were likely due to contamination from a second promoter construct. For this reason, these promoters were removed the analysis. Three samples were removed from the analysis, one on the basis of abnormal DNA staining, and two due to small sample sizes.

We calculated a 95% confidence interval around the mean fluorescence of the empty vectors (containing gfp, but no promoter), and excluded all promoters with a mean fluorescence less than this range from the analysis (below 2.26 fluorescence units). There is thus only a 2.5% chance that the GFP signal for any promoter included in the analysis is due to only to autofluorescence.

### Measuring variation in mRNA expression within a population

Our goal was to define a consistent metric of noise in mRNA expression that enabled comparison of genes with different mean expression levels (in other words, to decouple mean from variation in expression). We thus followed a method similar to that outlined by Newman et al. [24], in which noise is defined as the deviation from a sliding window of the median expression level versus the CV for each promoter. To more robustly estimate the deviation, we defined noise as the vertical deviation from a smoothed spline (6 degrees of freedom) that covered a running median of mean log expression versus CV of log expression (window of 15 data points); a smoothed spline is not subject to the small deviations that a running median is (Figure 1F). For simplicity, we refer to this deviation as noise in gene expression, or noise. We note that noise is homoscedastic across expression levels: mean expression level versus noise or the absolute value of noise gives no significant regression. This is not the case for two related metrics of noise based on vertical deviation from a smooth spline: if log mean expression versus CV of expression or mean log expression versus standard deviation of log expression are used, both result in highly expressed genes having extreme levels of noise (either very high or very low) (Figure 1B, 1C, 1E). In contrast, for the metric of noise we use, genes having very high expression are not more likely to have extreme levels of noise. In addition, there is no significant correlation of noise with mean expression level (rho = −0.035, p = 0.17, n = 1522). Lastly, our results are robust when using similar noise metrics (e.g. vertical deviation from the running median, Euclidean distance from the smoothed spline, or if different spline fits are used; see Text S1). The noise metric is a highly reliable measure; for separate measurements of two independent cultures grown in different growth media yields a Spearman's rho value of 0.58 (p<1e-120; Figure S3).

### Gene essentiality and growth phenotypes

Data on gene essentiality was taken from the PEC dataset [37]. Promoters were considered essential if they drove the expression of an essential gene or an operon containing an essential gene. For conservation, only the immediate downstream gene was taken into account.

### Gene conservation and horizontal transfer

Using data from Ragan et al. (2006), for each gene that appeared to have experienced horizontal transfer, we used the median value of the estimated phyletic depth at which the horizontal transfer occurred. We then selected those genes that had been acquired after the divergence of *E. coli* from *Haemophilus* (220 genes), *Buchnera* (170 genes), or *E. coli* CFT073 (42 genes), and used these sets to calculate the relationship in recently transferred genes between noise and gene conservation.

We calculated gene conservation using a reciprocal shortest distance strategy [69] to search for putative orthologues of *E. coli* genes in 105 fully sequenced gamma-proteobacteria or 58 alpha-proteobacteria [70]. We considered genes present in at least 30 out of 58 (>50%) fully sequenced alpha-proteobacterial taxa to have been acquired before the *E. coli* – alpha-proteobacteria divergence.

### Enrichment of functional classes for high or low noise promoters

Promoters were grouped by functional class according to the gene annotations for the immediate downstream gene, as outlined in MultiFun [48] into eight major categories: metabolism, information transfer, regulation, transport, cell process, cell structure, cellular location, and extra-chromosomal element; each major category contained up to eight subcategories. To test for the enrichment of low or high noise genes, for each major category, each subcategory was tested against the remaining genes in that major category for enrichment of promoters with higher or lower noise using a Wilcox rank sum test.

### mRNA abundances, half-lives, and expression ratios

Data on relative mRNA abundances and half-lives were taken from [55]. Data on relative mRNA expression levels (i.e. expression ratios) for 240 different conditions were taken from the *E. coli* Gene Expression Database (http://genexpdb.ou.edu/). These conditions were also grouped using hierarchical clustering into 18 clusters in which expression ratios were similar using the Lance-Williams formula as implemented by *hclust* in the R statistical package.

### Operon structure and sigma factor binding sites

Data on both operon structure and the binding sites of sigma factors was taken from RegulonDB (http://regulondb.ccg.unam.mx/).

### Noise in yeast orthologues

Orthologous genes in yeast were identified using a reciprocal best-hit analysis, with varying e-value cut-offs. The significance of the correlation, although low, is robust to changes in the stringency of the e-value cut-off (we note that as the stringency of this cutoff is increased, the number of orthologues decreases, necessarily decreasing the significance: e-20: rho = 0.2, p = 0.07; e-30: rho = 0.28, p = 0.02; e-40: rho = 0.26, p = 0.06; e-50: rho = 0.25, p = 0.11).

### Statistical analyses

Unless otherwise specified, all categorical comparisons were performed using a non-parametric two-sided Wilcox rank sum test and all reported correlations are non-parametric Spearman rank correlations. The p-values for the Spearman rank correlations were calculated using the default settings of the cor.test() function in R, which uses an asymptotic *t* approximation.

## Supporting Information

Figure S1 Gating methodology for FSC and SSC. Data for 100,000 cells was collected. From these cells, a subset of approximately 10,000 cells were selected from an elliptical gate (red) centered on the densest area of cells.
(PDF)

Figure S2 Repeatability of flow cytometry measurements of mean and standard deviation in gene expression. A. Repeatability of measurements of mean expression. Shown are measurements of two full biological replicates for 92 promoters measured using different settings on different flow cytometry machines in different laboratories, and with different filtering methods (red; r^2^ = 0.912) or on the same machine with the same settings and filtering methodology (black; r^2^ = 0.998). B. Repeatability of measurements of standard deviation in gene expression. Conditions and colors are identical to those in A. r^2^ = 0.509 and 0.922 for different and identical flow cytometry machines, respectively.
(PDF)

Figure S3 Repeatability of noise metric across growth conditions. Shown are two conditions of growth and the measured noise levels for all genes exhibiting mean fluorescence above background levels. The metric is highly consistent (rho = 0.58; p<1e-120).
(PDF)

Figure S4 Plasmid and chromosomally integrated promoters exhibit similar mean and variation in expression. We measured mean log expression and the coefficient of variation in expression for nine promoter-*gfp* fusions that were chromosomally integrated at the *attTn7* site and compared this to those found for the plasmid-based system. We found that the chromosomally integrated constructs exhibited good correlations with the plasmid-based system (rho = 0.85, p = 0.006; rho = 0.77, p = 0.016 for mean (left panel) and CV (right panel), respectively). We would expect there to be changes in either the mean or variation in expression if titration of transcription factors in the plasmid-based system had a large effect on regulation. It does not appear that this is the case. Although the chromosomal CV of *slp* appears smaller than when on the plasmid, some of this difference is likely due to the difficulty in accurately measuring the chromosomal CV for *slp*, as the fluorescence level is only slightly above the background fluorescence.
(PDF)

Figure S5 Full scatter plot of the relationship between gene conservation of non-essential genes and noise. The conservation level of 1334 non-essential genes is plotted against the phenotypic noise observed for each gene. As noted in the main text, this relationship is highly significant (Spearman's rho = −0.20, p = 4.75e-13). A non-parametric regression line fit using Thiel's incomplete method [71] is shown in red.
(PDF)

Figure S6 Conserved genes exhibit lower levels of noise. Four genes are shown as examples: *bhsA* (stress resistance), *glgS* (carbohydrate metabolism), *dnaK* (heat shock), and *lon* (protein degradation). *bhsA* and *glgS* both exhibit relatively high levels of noise, and are less well conserved; *dnaK* and *lon* exhibit low levels of noise and are almost perfectly conserved across gamma-proteobacteria.
(PDF)

Figure S7 Broad differences in gene expression noise between functional categories does not drive the negative correlation between gene conservation and noise. For each functional class containing more than 30 non-essential genes, the Spearman correlation between gene conservation and noise was calculated. The numbers in parentheses indicate the number of non-essential protein coding genes in that subcategory. For some subcategories, there is little variation in either conservation or noise; thus the correlation is not always strong. However, in nearly all cases, the correlation remains negative; those subcategories with p<0.05 are shaded in grey.
(PDF)

Figure S8 There is no relationship between standard deviation in gene expression across environments and noise in expression. Promoters were binned according to the observed standard deviation in gene expression across environments. Regardless of whether or how binning was performed, no significant relationship between the standard deviation in gene expression across environments and the level of noise in expression could be found. This contrasts strongly with previous results from previous studies in yeast.
(PDF)

Text S1 Supplementary information containing further details of the analysis and discussion.
(DOC)

Dataset S1 Mean, median, standard deviation, and noise values for each promoter. The first column lists the name of the downstream gene; the next four columns list the mean and standard deviation of the fluorescence values for the log-transformed and original data, respectively. The sixth column lists the noise statistic for each gene.
(XLS)

Dataset S2 Simplified code (in R) that was used to process the raw FACS data.
(R) Click here for additional data file.
