# Correction: A Genome-Wide Analysis of Promoter-Mediated Phenotypic Noise in *Escherichia coli*

**DOI:** 10.1371/annotation/73cf6e53-2141-4918-926b-8d07b073884d

**Published:** 2012-05-18

**Authors:** Olin K. Silander, Nela Nikolic, Alon Zaslaver, Anat Bren, Ilya Kikoin, Uri Alon, Martin Ackermann

Figure S6 is incorrectly a duplicate of Figure S7. The correct Figure S6 can be viewed here:

[^] 

