# Correction: A Genome-Wide Analysis of Promoter-Mediated Phenotypic Noise in *Escherichia coli*

**DOI:** 10.1371/annotation/0ddf7d6d-9118-46b9-a15a-85673e7fc29e

**Published:** 2012-05-18

**Authors:** Olin K. Silander, Nela Nikolic, Alon Zaslaver, Anat Bren, Ilya Kikoin, Uri Alon, Martin Ackermann

Figure S7 is incorrectly a duplicate of Figure S6. The correct Figure S7 can be viewed here:

[^]

