# Supplementary figures and images for: A Genome-Wide Analysis of Promoter-Mediated Phenotypic Noise in Escherichia coli
Source: PLoS Genet. 2012 Jan 19;8(1):e1002443. doi: 10.1371/journal.pgen.1002443 (PMC3261926; doi:10.1371/journal.pgen.1002443)

Log10 side scatter

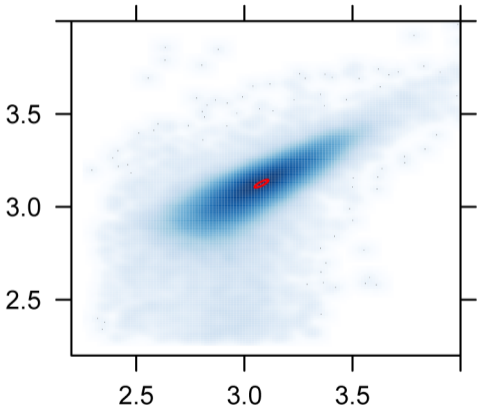

Log10 forward scatter

Supplement: Figure S1 — Gating methodology for FSC and SSC. Data for 100,000 cells was collected. From these cells, a subset of approximately 10,000 cells were selected from an elliptical gate (red) centered on the densest area of cells. (PDF) [file pgen.1002443.s001.pdf]

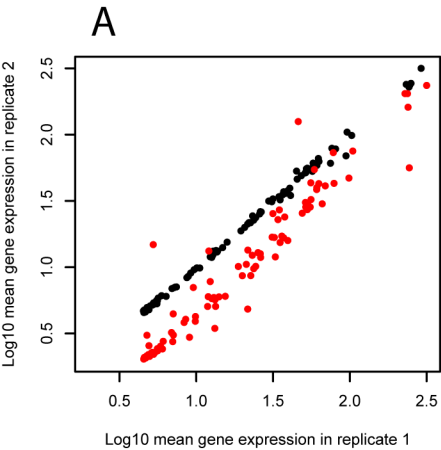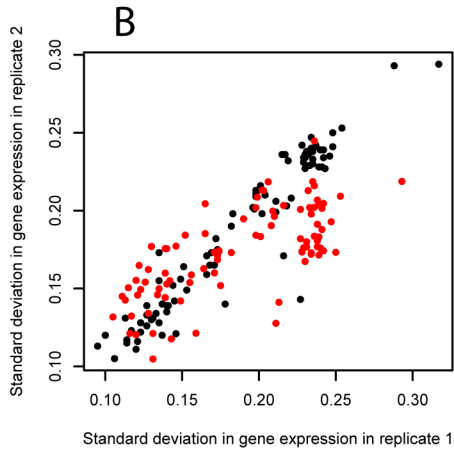

Supplement: Figure S2 — Repeatability of flow cytometry measurements of mean and standard deviation in gene expression. A. Repeatability of measurements of mean expression. Shown are measurements of two full biological replicates for 92 promoters measured using different settings on different flow cytometry machines in different laboratories, and with different filtering methods (red; r2 = 0.912) or on the same machine with the same settings and filtering methodology (black; r2 = 0.998). B. Repeatability of measurements of standard deviation in gene expression. Conditions and colors are identical to those in A. r2 = 0.509 and 0.922 for different and identical flow cytometry machines, respectively. (PDF) [file pgen.1002443.s002.pdf]

Scaled noise in 0.25 MIC ciprofloxacin

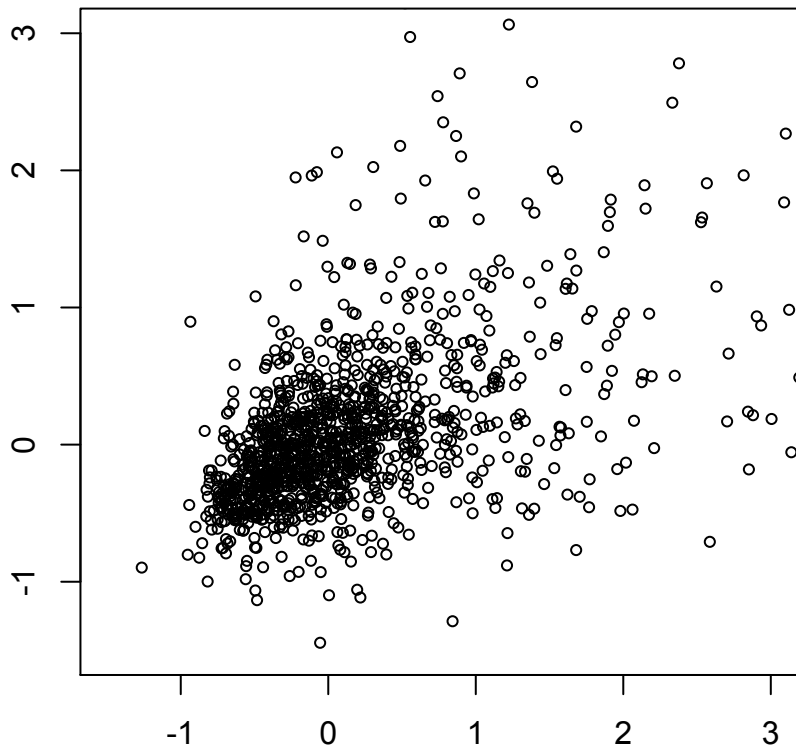

Scaled noise in minimal glucose media

Supplement: Figure S3 — Repeatability of noise metric across growth conditions. Shown are two conditions of growth and the measured noise levels for all genes exhibiting mean fluorescence above background levels. The metric is highly consistent (rho = 0.58; p<1e-120). (PDF) [file pgen.1002443.s003.pdf]

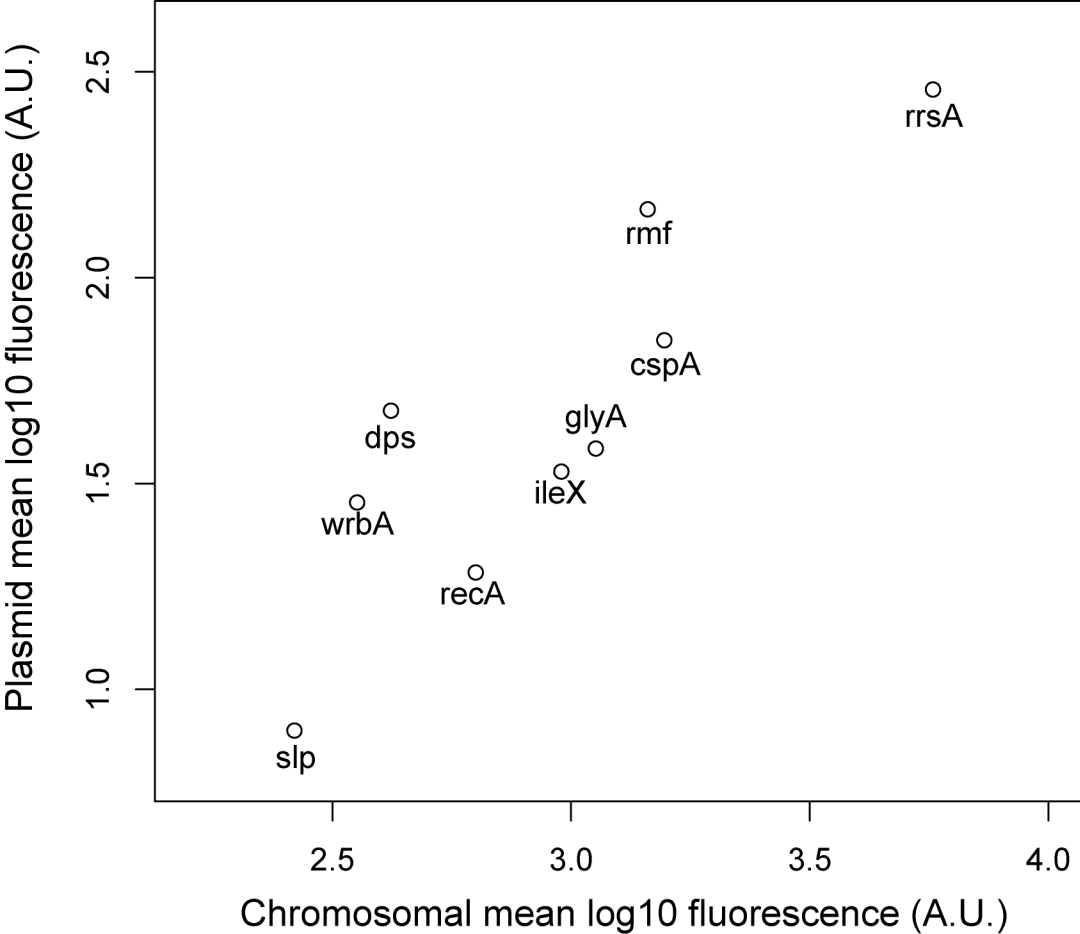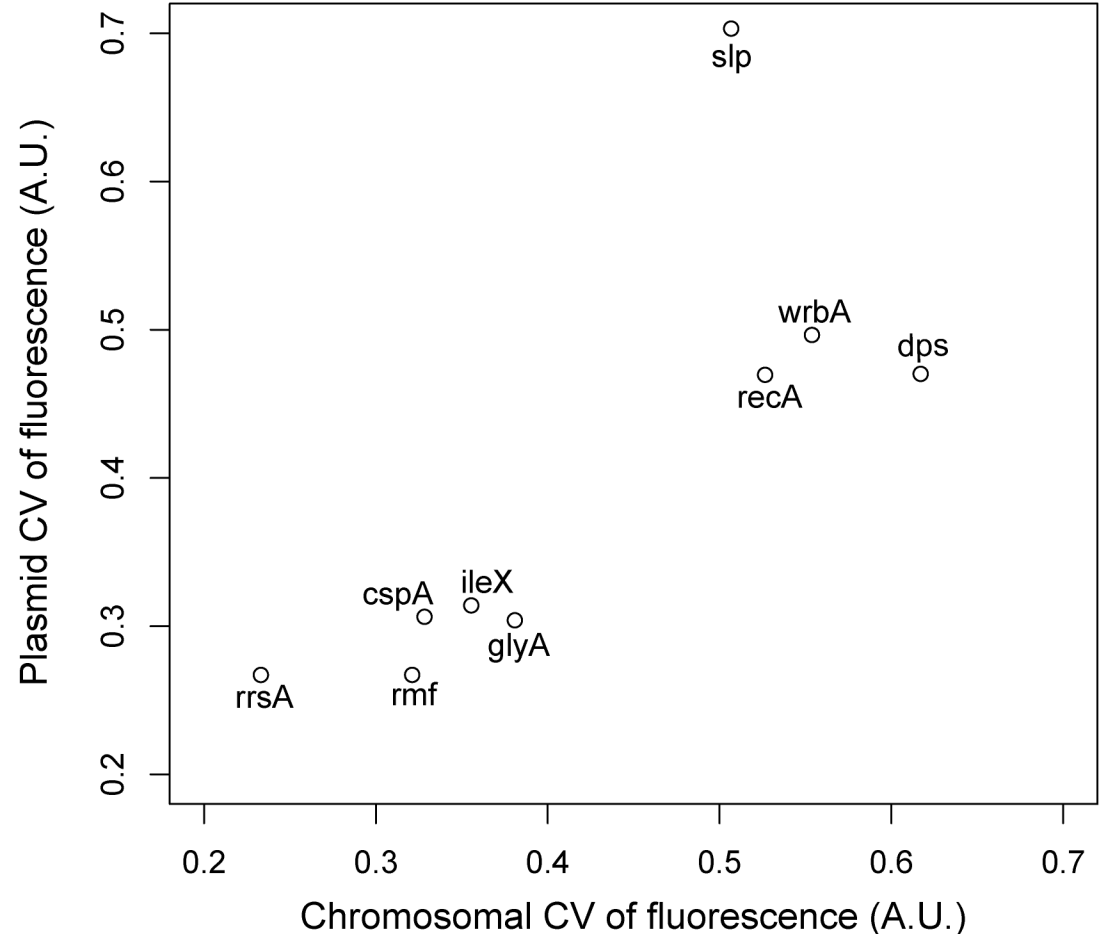

Supplement: Figure S4 — Plasmid and chromosomally integrated promoters exhibit similar mean and variation in expression. We measured mean log expression and the coefficient of variation in expression for nine promoter-gfp fusions that were chromosomally integrated at the attTn7 site and compared this to those found for the plasmid-based system. We found that the chromosomally integrated constructs exhibited good correlations with the plasmid-based system (rho = 0.85, p = 0.006; rho = 0.77, p = 0.016 for mean (left panel) and CV (right panel), respectively). We would expect there to be changes in either the mean or variation in expression if titration of transcription factors in the plasmid-based system had a large effect on regulation. It does not appear that this is the case. Although the chromosomal CV of slp appears smaller than when on the plasmid, some of this difference is likely due to the difficulty in accurately measuring the chromosomal CV for slp, as the fluorescence level is only slightly above the background fluorescence. (PDF) [file pgen.1002443.s004.pdf]

Phenotypic noise

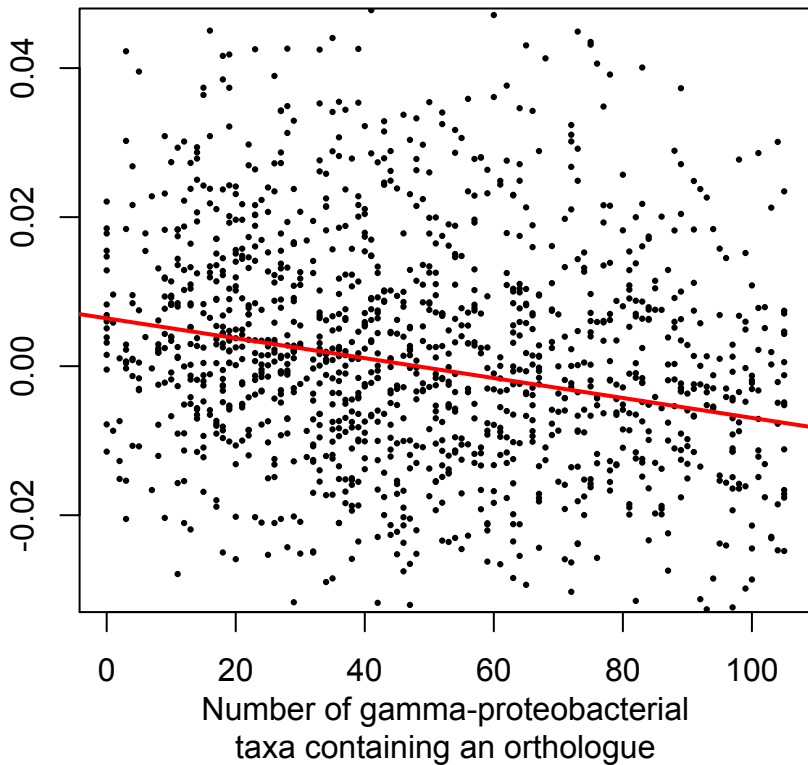

Supplement: Figure S5 — Full scatter plot of the relationship between gene conservation of non-essential genes and noise. The conservation level of 1334 non-essential genes is plotted against the phenotypic noise observed for each gene. As noted in the main text, this relationship is highly significant (Spearman's rho = −0.20, p = 4.75e-13). A non-parametric regression line fit using Thiel's incomplete method [71] is shown in red. (PDF) [file pgen.1002443.s005.pdf]

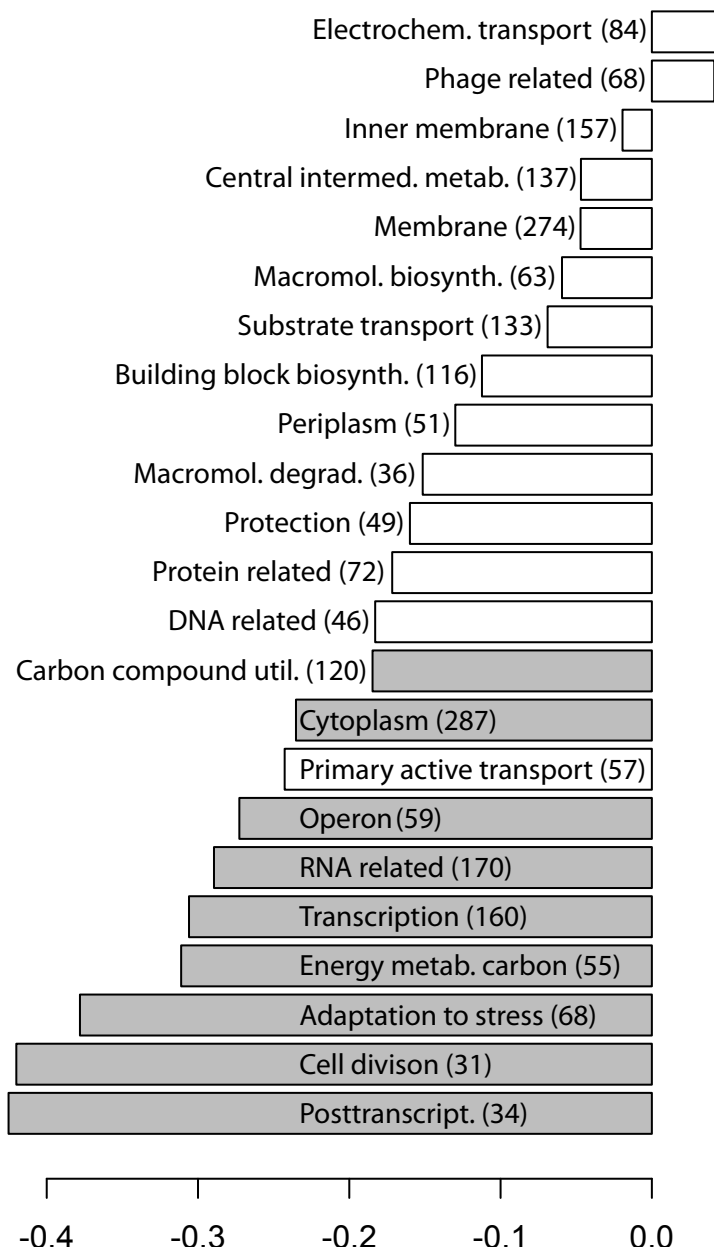

Correlation between noise and conservation  
(Spearman's rho)

Supplement: Figure S6 — Conserved genes exhibit lower levels of noise. Four genes are shown as examples: bhsA (stress resistance), glgS (carbohydrate metabolism), dnaK (heat shock), and lon (protein degradation). bhsA and glgS both exhibit relatively high levels of noise, and are less well conserved; dnaK and lon exhibit low levels of noise and are almost perfectly conserved across gamma-proteobacteria. (PDF) [file pgen.1002443.s006.pdf]

Kernel Density

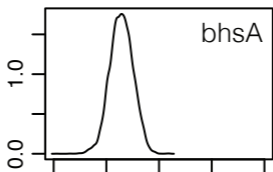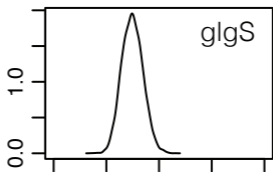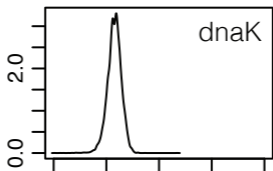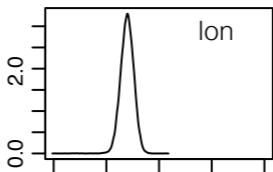

0 1 2 3 4  
Fluorescence (A.U.)

Supplement: Figure S7 — Broad differences in gene expression noise between functional categories does not drive the negative correlation between gene conservation and noise. For each functional class containing more than 30 non-essential genes, the Spearman correlation between gene conservation and noise was calculated. The numbers in parentheses indicate the number of non-essential protein coding genes in that subcategory. For some subcategories, there is little variation in either conservation or noise; thus the correlation is not always strong. However, in nearly all cases, the correlation remains negative; those subcategories with p<0.05 are shaded in grey. (PDF) [file pgen.1002443.s007.pdf]

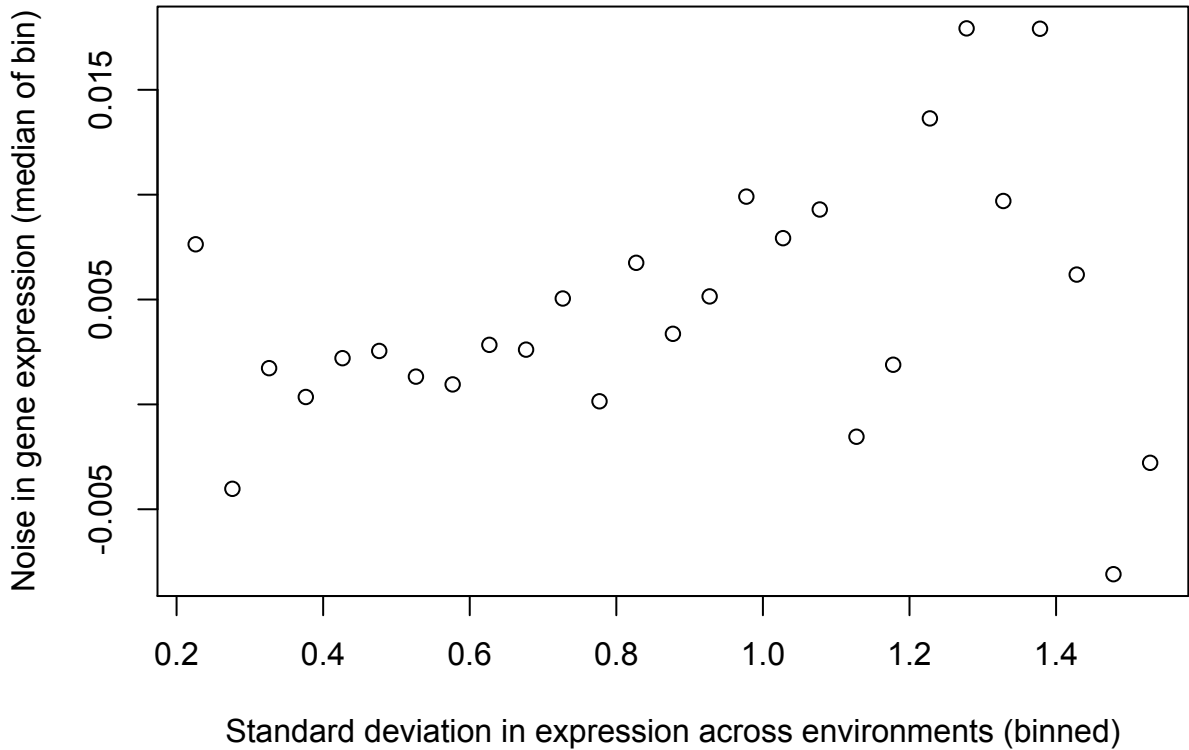

Supplement: Figure S8 — There is no relationship between standard deviation in gene expression across environments and noise in expression. Promoters were binned according to the observed standard deviation in gene expression across environments. Regardless of whether or how binning was performed, no significant relationship between the standard deviation in gene expression across environments and the level of noise in expression could be found. This contrasts strongly with previous results from previous studies in yeast. (PDF) [file pgen.1002443.s008.pdf]
