# Supplementary material for: A Genome-Wide Analysis of Promoter-Mediated Phenotypic Noise in Escherichia coli
Source: PLoS Genet. 2012 Jan 19;8(1):e1002443. doi: 10.1371/journal.pgen.1002443 (PMC3261926; doi:10.1371/journal.pgen.1002443)
Supplement: Text S1 — Supplementary information containing further details of the analysis and discussion. (DOC) [file pgen.1002443.s009.doc]

**SUPPLEMENTARY TEXT**

**1. Testing the assumptions of our approach**

**Use of a plasmid-based system to determine relative noise levels**

We used a plasmid-based system for our assay of promoter-mediated noise. As pointed out in the text, this is an indirect and qualitative measure of the true promoter-mediated noise, as the gene has been removed from the chromosome and placed on a low-copy number plasmid (3-5 copies per cell); this affects both intrinsic and extrinsic noise (see the section below for more details). The plasmid is tightly regulated, but the numbers of plasmids per chromosomal copy in the cell may fluctuate. These certainly affect the level of mRNA and protein in a cell, but this effect should be similar for all plasmid constructs. We have no reason to expect that the amount of cell-to-cell variation in plasmid number depends on which promoter resides on the plasmid (especially after correcting for mean expression level), so this factor should not play a role in determining our relative transcriptional noise levels. In particular, correcting for mean expression level should eliminate any effects that may affect variation because of toxic levels of GFP in the cell (see below for more).

As noted in the main text, we used a small set of chromosomal integrations to validate our measurements using the plasmid-based assay. We find that the chromosomal constructs exhibit very similar characteristic mean and noise levels for each promoter (rho=0.77, p=0.014; rho=0.78, p=0.012 for mean and CV, respectively; **Fig. S4**). Thus, our system, despite being plasmid-based, accurately reflects what is observed in a chromosomal context.

It is possible that strong promoters causing high levels of GFP may impose stress. However, the levels used for these experiments are not so high that they should cause significant disruption in growth. Measurements in *Salmonella* have shown that when the number of GFP molecules are approximately ten-fold higher than the number of ribosomes (i.e. ~500,000 GFP molecules), doubling time slows only by approximately 10% . Because we use native *E. coli* promoters, we expect that the number of GFP molecules within the cell will almost always be less than this number, even though the plasmids themselves are present in multiple copies. Additionally, stress due to the number of GFP molecules are unlikely to differ for cells with the same level of GFP fluorescence, so the fact that we see consistent differences in noise after having corrected for mean expression level implies that differences in variation between promoters are not driven by cellular stress due to GFP production.

### GFP fluorescence correlates with mRNA levels

To critically evaluate the hypothesis that promoter regions alone affect transcriptional processes to determine gene expression levels in a manner similar to when such promoters are present in their native locations, we compared the fluorescence levels that we found to two studies that reported mean mRNA levels using microarray hybridization with a genomic DNA control as well as a recent study which used RNA-seq . All of these studies measure mRNA levels during growth in minimal or rich medium, and directly measure transcript levels, which are at a steady-state determined by transcriptional and degradation processes. In our experimental system, degradation is not regulated in a normal manner, which will decrease the strength of the correlation. In addition, we infer transcriptional processes indirectly through measuring protein concentrations. Despite this, in all cases, the mean GFP expression levels that we measure directly are highly correlated with directly measured levels of mRNA (rho=0.44, p=1.9e-61; rho=0.34, p=2.1e-29; rho=0.48, p=4.9e-21 respectively). These correlations approach those found between these studies (rho=0.54-0.68), suggesting that our data set provides a good description of transcriptional processes.

As a more relevant comparison, the correlation of native mRNA transcript levels measured through RNA-seq, and non-native YFP fusion mRNAs measured by FISH, was found previously to be 0.51 . This value is very close to what we find.

Although our measurements do not fully correlate with directly measured mRNA levels, our aim is not to design an experimental system that allows us to infer mRNA transcript levels from measured GFP levels. The correlation that we find between directly measured mRNA levels and those inferred from GFP fluorescence indicates that removing the promoter from native chromosomal context and regulation of degradation, measuring the fluorescence of promoter-*gfp* fusions reveals some information, but not all, about the true expression level of a gene. Similarly, our method of inference (using a simple, straightforward plasmid-based technique in which promoter regions are cloned upstream of GFP) allows us to measure some, but not all, of the variation in mRNA expression for each gene (e.g. **Fig. S4**).

We find a much lower correlation with measured protein levels (rho=0.24, half the strength of the mRNA correlation from the same study). This is expected, as our experimental system does not include any aspect of post-transcriptional regulation, and thus the mean level of GFP largely reflects the processes of transcription, and not translation or mRNA and protein degradation.

Finally, we have excluded genes with expression levels below our detection limit from the analysis, and this may cause a bias in the types of genes that are included. Although this prevents us from making any inferences on whether there are promoter-mediated effects on noise for this subset of weakly expressed genes, this should not affect our conclusions for more strongly expressed genes concerning the relationships between gene importance, gene regulation, or gene function and noise.

**Noise metric**

As our principal aims is to infer whether variation in gene expression can be changed independently from mean expression (decoupled), we corrected our measure of variation for mean expression using the vertical deviation from a smooth spline of mean expression versus the coefficient of variation in log expression as our metric of noise (see Supplementary data file). Analyses using a similar metric, the Euclidean distance to the spline, yielded similar results. In addition, changing the fit of the spline (12 degrees of freedom and a running median window of 21 points) or using solely the running median (window of 21 points) does not qualitatively affect the results. The correlation of noise with conservation, among non-essential genes, remains strong (rho=-0.19, p=8.1e-13; rho=-0.18, p=4.4e-11, respectively). Two other mean-corrected noise metrics, the vertical deviation of mean expression versus the coefficient of variation of expression (**Fig. 1C**); and mean log expression versus the standard deviation of log expression (**Fig. 1E**), are less well suited as a noise metric because their spread increases strongly with increasing expression level; in other words, they are heteroscedastic.

### Contributions of extrinsic and intrinsic noise to total noise

In addition to differences in transcriptional regulation, changes in plasmid copy number, measurement error, and background fluorescence all contribute to the measurements of variation. While measurement error and background fluorescence generally add experimental variation to measured GFP levels, the presence of multiple plasmid copies affects intrinsic and extrinsic noise levels in a specific manner. Due to mRNA being transcribed from multiple plasmid copies, fluctuations in transcription because of transcription factor binding, polymerase binding, and mRNA degradation are lower. For this reason, the fraction of noise explained by intrinsic noise will be decreased. In addition, we have excluded all genes with low levels of expression; these genes generally have the highest fraction of intrinsic noise . Fluctuations between cells in plasmid copy-number will increase the amount of extrinsic noise (for definitions of intrinsic and extrinsic noise see ); however, as mentioned above, the increase in extrinsic noise due to copy-number variation should not depend on which promoter resides on the plasmid. Thus, additional extrinsic sources of variation should be nearly identical for transcripts expressed at a specific level: if two promoters differ consistently in the cell-to-cell variation, this difference in implies that on some level, the promoter sequence itself controls the level of extrinsic noise.

Elowitz et al. (2002) note that extrinsic noise is smaller in cells with a chromosomal copy of a gene (lacI) than in cells with a plasmid-borne copy of the gene. It is likely that this is not an exceptional case, and that it is true for most genes, probably as a result of fluctuations in copy number of the plasmid. Thus, our system likely exhibits increased levels of extrinsic noise. However, our analysis is based on *relative* noise levels that have been corrected for mean expression. This correction, and the basis on relative noise levels and not quantitative noise levels, allow us to make meaningful statistical inferences on how transcriptional noise levels change among promoters driving genes of different function, conservation, or recent transfer. This point is critical for our analysis: our aim is not to parameterize mechanisms or to quantify transcriptional noise, but to be able to make meaningful inferences about which genes have relatively higher or lower levels of noise. Thus, even though extrinsic noise levels are likely to be globally increased for our system, as it is plasmid based, this increase should affect all genes expressed at the same level in an equal manner. Our correction for mean expression level ensures that variation in plasmid number is not an issue for our analysis.

Although some data suggests that intrinsic noise dominates transcription , this depends on the context. One example is in the expression of the *lac* operon: at certain induction levels, populations of cells exhibit bimodal expression levels, in which part of the population expresses *lacZYA* at high levels, further inhibiting LacI repression of *lacZYA*, while a second part of the population expresses *lacZYA* at very low levels, causing LacI to continue acting as a repressor of *lacZYA*. In this case, transcriptional noise in *lacZYA* expression is almost exclusively extrinsic. Thus, there may be many instances in which transcriptional control is not dominated by intrinsic noise. As our system is largely insensitive to differences in intrinsic noise, but we find consistent differences in noise, for example between functional categories, this result must be due to the fact that there are meaningful differences between genes in their extrinsic transcriptional noise. However, intrinsic noise may have an additional effect in these cases.

### 2. Alternative explanations for our findings

### Effects of growth selection on noise

### We have claimed that the decrease in variation that we observe in essential genes is due to these genes having lower levels of transcriptional noise. An alternative explanation is that this is instead due to a selection effect that occurs during growth – those cells with very high or very low levels of essential genes fail to grow as quickly, thus biasing the set of cells that we measure such that they have lower levels of variation in transcription. Two points speak against this possibility. First, we have filtered our cells extensively, so that only 10% of all measured cells were included to infer noise levels; all of these cells appeared physiologically similar and were likely to have been growing at similar rates. Previous studies have shown that cells that grow slowly tend to have lower protein levels , such that a decrease in growth rate from 2 doublings per hour to 1 doubling per hour causes an approximate 30% decrease in optical density per cell ; this should manifest as decreased side scatter. When we gated on side scatter, the gates we used varied by approximately 10% of the SSC value (Fig. S1). As some of this variation is a result of measurement error alone, and not physiological differences, we expect that the differences in optical density (and therefore protein levels) are even less. Thus, cells within the gated populations should not have significant differences in growth rates, and all cells, regardless of their level of GFP, should be equally represented.

### On the other hand, if the plasmid-based promoters affect the stoichiometry of transcription factors that control essential genes, this may cause some cells to be stressed, thereby increasing the variation in physiological state. However, this would only increase the size of the gate, and thus increase the variation in GFP expression for these promoters, which is the opposite of the pattern that we observe. Secondly, we find a strong relationship between noise and conservation for non-essential genes, and this pattern manifests across different functional categories. These genes, all of which are non-essential, should be less affected by any bias in growth rates than essential genes (we have shown previously that there is only a slight negative correlation between growth rates and conservation level ). Again, this implies that selection over evolutionary time due to functional importance, and not selection during growth in the culture, drives the differences in noise between genes.

### The absence of an association between promoter-mediated noise and expression plasticity in *E. coli* is not due to differences in data quality

The data on noise for yeast and *E. coli* differ substantially: the *E. coli* data largely excludes noise arising from gene-specific post transcriptional mechanisms, and there may be additional differences in accuracy due to the difference in size and other factors between yeast and *E. coli*. The expression data on which the expression plasticity is based on differ substantially, as different sets of growth conditions were used for each organism. Additionally, expression changes may generally be less substantial in *E. coli* than in yeast, decreasing the likelihood of finding significant associations. However, we do not think that any of these explanations can fully explain what we observe (**Fig. S5**). In particular, we find that the strength of the correlations between *change* in the expression and noise are similar for both yeast and *E. coli*. That is, for 125 pairs out of the 173 total pairs of growth conditions for yeast, there are negative or positive correlations between noise and expression *change* with (i.e. rho less than -0.1 or greater than 0.1). In *E. coli*, the correlations are slightly less strong, appearing for 96 out of the 240 total pairs of growth conditions. Testing for an association between noise and *expression plasticity*, the picture changes (for a discussion about the difference between ‘expression change’ and ‘expression plasticity,’ see the last paragraph of this section). In yeast, 76 out of 173 pairs of conditions have positive correlations between noise and expression plasticity (i.e. with rho > 0.1); none have negative correlations. In *E. coli*, only 5 out of 241 pairs of growth conditions have similarly strong positive correlations between noise and expression plasticity. The fact that a large number of the correlations between expression change and noise are significant for both *E. coli* and yeast suggests that the data sets do not differ substantially in their quality. Thus, we suggest the lack of a correlation between expression plasticity and noise is not due simply to the noise and expression data in *E. coli* being qualitatively less accurate.

In addition to performing an analysis on expression plasticity (median change in expression across environments), we analyzed the standard deviation in expression across environments, as in . We binned this data to look for any indication that noise is dependent on the standard deviation of expression. Again, we did not find any clear relationship (**Fig. S8**).

Perhaps related to this phenomenon are the functional differences between yeast and *E. coli* in terms of which genes exhibit the highest expression plasticity. While essential genes are generally expressed at higher levels for both yeast and *E. coli* (Wilcox rank sum for expression level versus essentiality, p=3e-5 and 2e-24, respectively), only in *E. coli* do essential genes have higher expression plasticity (p=5e-6). This seems to support the lack of a connection between expression plasticity and noise in *E. coli*: despite essential genes having significantly higher expression plasticity, they have significantly lower levels of noise. Interestingly, in yeast, essential genes have slightly lower expression plasticity (p=0.01), as well as lower levels of noise.

As noted above, some pairs of experimental conditions exhibit significant positive or negative correlations between gene expression *change* (whether a gene increases or decreases in a certain condition) and noise. For example, genes that are up-regulated (or are at higher relative concentrations) after 20 min. treatment with kanamycin tend to exhibit low levels of noise (e.g. cell division genes, heat shock response genes), while genes that are down-regulated show high levels of noise. In contrast, genes that are up-regulated after 20 min. treatment with norfloxacin show high levels of noise (SOS genes), while genes that are down-regulated show low levels. We emphasize that this is not what we would expect if the expression plasticity of a gene determines its level of noise – we would expect that genes that are *either* up- *or* down-regulated in response to environmental signals would be noisy. Although we cannot provide a full explanation for the correlations between noise and expression change at this point, we propose that it may simply be due to certain functional classes being up- or down-regulated.

**3. Comparisons to other studies**

### Correlations with other data sets

The correlation we find between our noise data and a second data set for which we calculated protein noise is significant, but low (rho=0.12, p=0.02, n=329) (however, we cannot assess the reliability of this metric of protein noise as we do not have data on replicate measurements). This low correlation may be caused by several phenomena. First, the measurements for the two datasets were not taken during the same stages of growth: our measurements occurred during early exponential phase, while those of Taniguchi et al. occurred during late exponential phase (11-12 hours of growth at 30C to OD 0.1-0.5). Second, constructs in the second study were present in the native chromosomal context, which may have effects on accessibility, transcription rates or bursting and other intrinsic noise sources, such as copy number variation due to proximity to the ori or terminus. Third, the constructs in contained the native ribosomal binding site and 5’ mRNA sequence, both of which significantly affect ribosomal binding, and may also affect noise . Fourth, Taniguchi et al. utilized translational fusion constructs, which in some cases may affect the behavior of the protein, especially degradation. Different culture conditions (e.g. solid vs. liquid media) may have significantly affected the noise levels of different genes. No filtering based on cell size or physiology was done, in contrast to our own data, which were stringently gated on such traits. Finally, Taniguchi et al. quantified noise using microscopy data, while we have measure noise using FACS measurements; systematic differences or biases between these two methods may weaken the correlation between the two datasets.

We find a very low correlation between mRNA noise from the Taniguchi dataset, corrected for mean expression, and the promoter-mediated noise that we measure (rho=0.082, p=0.52, n=63). However, we also find no strong correlation between mRNA noise and protein noise (also corrected for mean expression) within the Taniguchi data set (rho=0.089, p=0.30, n=137). This low correlation may partially be due to the mRNA data in being less accurate, or to the mRNA noise metric being less accurate, as mRNA variation was measured for a small number of genes.

Despite the differences between the two data sets on promoter-mediated noise and protein noise, both correlate with functional traits in ways that are very similar, including with essential genes, conserved genes, and genes which change expression under certain conditions (e.g. kanamycin treatment; see above); as well as an absence of a correlation with expression plasticity. This suggests that both studies capture important aspects of noise, although not necessarily identical aspects. It would be informative to have a similar genome-wide study of noise at the extrinsic post-transcriptional level, as it is not possible to infer this from the current datasets.

**References**

1. Bollenbach T, Kishony R (2011) Resolution of Gene Regulatory Conflicts Caused by Combinations of Antibiotics. Molecular Cell 42: 413-425.

2. Wendland M, Bumann D (2002) Optimization of GFP levels for analyzing Salmonella gene expression during an infection. Febs Letters 521: 105-108.

3. Khodursky AB, Peter BJ, Cozzarelli NR, Botstein D, Brown PO, et al. (2000) DNA microarray analysis of gene expression in response to physiological and genetic changes that affect tryptophan metabolism in Escherichia coli. Proc Natl Acad Sci U S A 97: 12170-12175.

4. Bernstein JA, Khodursky AB, Lin PH, Lin-Chao S, Cohen SN (2002) Global analysis of mRNA decay and abundance in Escherichia coli at single-gene resolution using two-color fluorescent DNA microarrays. Proceedings of the National Academy of Sciences of the United States of America 99: 9697-9702.

5. Taniguchi Y, Choi PJ, Li G-W, Chen H, Babu M, et al. (2010) Quantifying E. coli Proteome and Transcriptome with Single-Molecule Sensitivity in Single Cells. Science 329: 533-538.

6. Elowitz MB, Levine AJ, Siggia ED, Swain PS (2002) Stochastic gene expression in a single cell. Science 297: 1183-1186.

7. Golding I, Paulsson J, Zawilski SM, Cox EC (2005) Real-time kinetics of gene activity in individual bacteria. Cell 123: 1025-1036.

8. Scott M, Gunderson CW, Mateescu EM, Zhang ZG, Hwa T (2010) Interdependence of Cell Growth and Gene Expression: Origins and Consequences. Science 330: 1099-1102.

9. Schaechter M, Maaloe O, Kjeldgaard NO (1958) Dependency on Medium and Temperature of Cell Size and Chemical Composition during Balanced Growth of Salmonella-Typhimurium. Journal of General Microbiology 19: 592-606.

10. Silander OK, Ackermann M (2009) The constancy of gene conservation across divergent bacterial orders. BMC Research Notes 2: 2.

11. Newman JRS, Ghaemmaghami S, Ihmels J, Breslow DK, Noble M, et al. (2006) Single-cell proteomic analysis of S-cerevisiae reveals the architecture of biological noise. Nature 441: 840-846.

12. Sangurdekar D, Srienc F, Khodursky A (2006) A classification based framework for quantitative description of large-scale microarray data. Genome biology 7: R32.

13. Ozbudak EM, Thattai M, Kurtser I, Grossman AD, van Oudenaarden A (2002) Regulation of noise in the expression of a single gene. Nature Genetics 31: 69-73.
