# Supplementary figures and images for: Correction: A Genome-Wide Analysis of Promoter-Mediated Phenotypic Noise in Escherichia coli
Source: PLoS Genet. 2012 May 18;8(5):10.1371/annotation/73cf6e53-2141-4918-926b-8d07b073884d. doi: 10.1371/annotation/73cf6e53-2141-4918-926b-8d07b073884d (PMC3368960; doi:10.1371/annotation/73cf6e53-2141-4918-926b-8d07b073884d)

Kernel Density

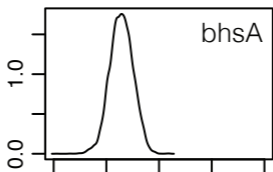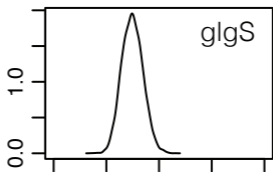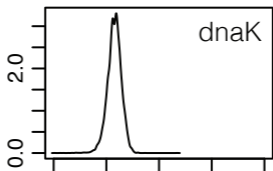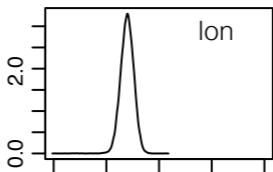

0 1 2 3 4  
Fluorescence (A.U.)

Supplement: Supplementary file 1 [file pgen.73cf6e53-2141-4918-926b-8d07b073884d.s001.pdf]
