# Supplementary figures and images for: Correction: A Genome-Wide Analysis of Promoter-Mediated Phenotypic Noise in Escherichia coli
Source: PLoS Genet. 2012 May 18;8(5):10.1371/annotation/0ddf7d6d-9118-46b9-a15a-85673e7fc29e. doi: 10.1371/annotation/0ddf7d6d-9118-46b9-a15a-85673e7fc29e (PMC3368961; doi:10.1371/annotation/0ddf7d6d-9118-46b9-a15a-85673e7fc29e)

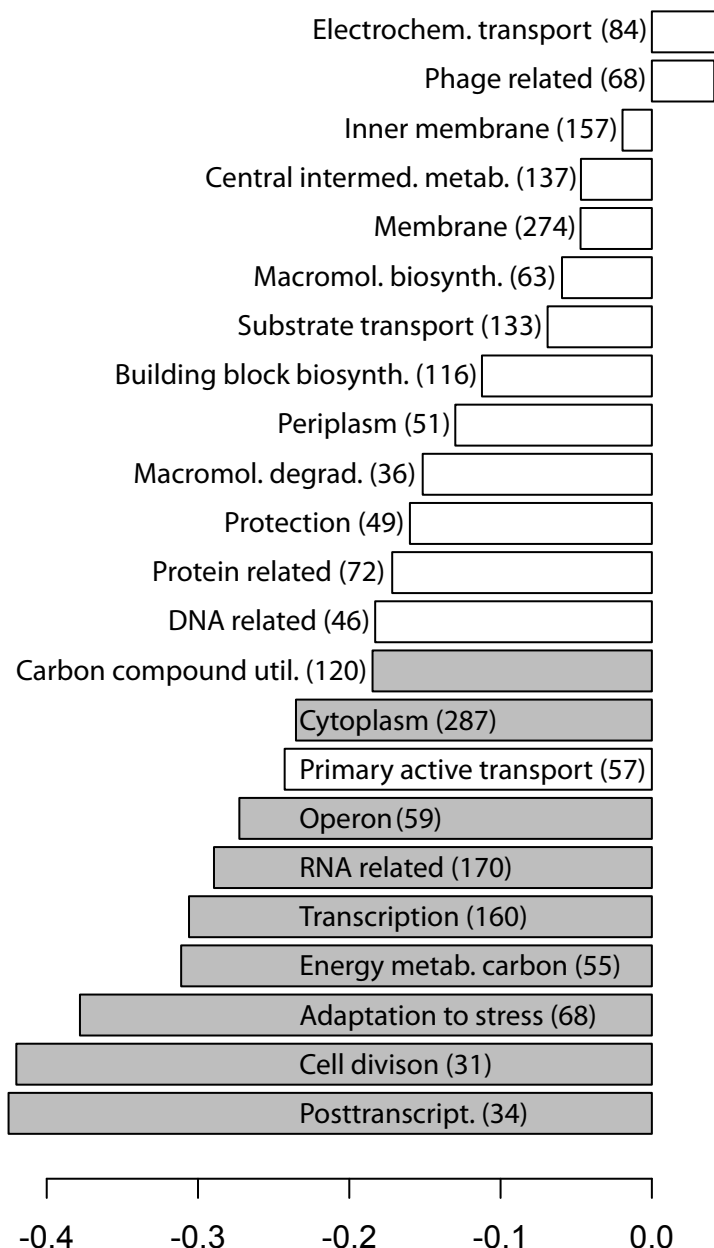

Correlation between noise and conservation  
(Spearman's rho)

Supplement: Supplementary file 1 [file pgen.0ddf7d6d-9118-46b9-a15a-85673e7fc29e.s001.pdf]
